# The Unexpected Selectivity Switching from Mitochondria to Lysosome in a D-π-A Cyanine Dye

**DOI:** 10.3390/bios12070504

**Published:** 2022-07-10

**Authors:** Chathura S. Abeywickrama, Hannah J. Baumann, Keti A. Bertman, Brian Corbin, Yi Pang

**Affiliations:** 1 Department of Structural Biology, St Jude Children’s Research Hospital, Memphis, TN 38105, USA; 2 Department of Chemistry, The University of Akron, Akron, OH 44325, USA; hjbaumann93@gmail.com (H.J.B.); kgb19@uakron.edu (K.A.B.); bpc32@uakron.edu (B.C.); 3 Maurice Morton Institute of Polymer Science, The University of Akron, Akron, OH 44325, USA

**Keywords:** donor-π-acceptor molecules, cyanine dyes, fluorescence confocal microscopy, lysosome selectivity, photostability

## Abstract

Two interesting benzothizolium-based D-π-A type hemicyanine dyes (**3a**–**3b**) with a diphenylamine (-NPh_2_) donor group were evaluated for fluorescence confocal microscopy imaging ability in live cells (MO3.13, NHLF). In sharp contrast to previously reported D-π-A dyes with alkyl amine donor (-NR_2_) groups (**1**), **3a** and **3b** exhibited significantly different photophysical properties and organelle selectivity. Probes **3a** and **3b** were nearly non-fluorescent in many polar and non-polar solvents but exhibited a bright red fluorescence (λ_em_ ≈ 630–640 nm) in stained MO3.13 and NHLF with very low probe concentrations (i.e., 200 nM). Fluorescence confocal microscopy-based co-localization studies revealed excellent lysosome selectivity from the probes **3a**–**3b**, which is in sharp contrast to previously reported D-π-A type benzothiazolium dyes (**1**) with an alkyl amine donor group (-NR_2_) (exhibiting selectivity towards cellular mitochondria). The photostability of probe **3** was found to be dependent on the substituent (R’) attached to the quaternary nitrogen atom in the cyanine dye structure. The observed donor-dependent selectivity switching phenomenon can be highly useful in designing novel organelle-targeted fluorescent probes for live-cell imaging applications.

## 1. Introduction

Cyanine dyes have been widely used as fluorescent probes for numerous bioimaging applications due to their structural tunability, high biocompatibility, and excellent photophysical proprieties [1,2,3,4,5,6,7,8,9]. The development of fluorescent dyes with enhanced Stokes’ shift (i.e., Δλ ≈ 50 nm or higher) is one of the greatest advancements in developing fluorescent imaging probes, as their use can significantly reduce the background interferences from excitation photons while improving signal to noise ratio [10,11,12,13,14,15]. Excited-state intramolecular charge transfer (ESIPT) and Intra-molecular charge transfer (ICT) are two of the most widely used photophysical pathways that have been utilized for designing fluorescent dyes with large Stokes’ shifts [16,17,18,19,20,21,22,23]. Intra-molecular charge transfer (ICT) based fluorescent probes have gained significant attention in recent years due to their high sensitivity toward the changes in the environment (i.e., viscosity, polarity, temperature, pH, etc.) [11,24,25,26,27,28,29,30]. In responding to such environmental changes, ICT-based probes often exhibit distinguishable changes in their optical properties (i.e., shifts in emission or absorption maxima, changes in fluorescence intensity, fluorescence turn ON/OFF, etc.), which is a great advantage in various detection applications.

**Scheme 1:**
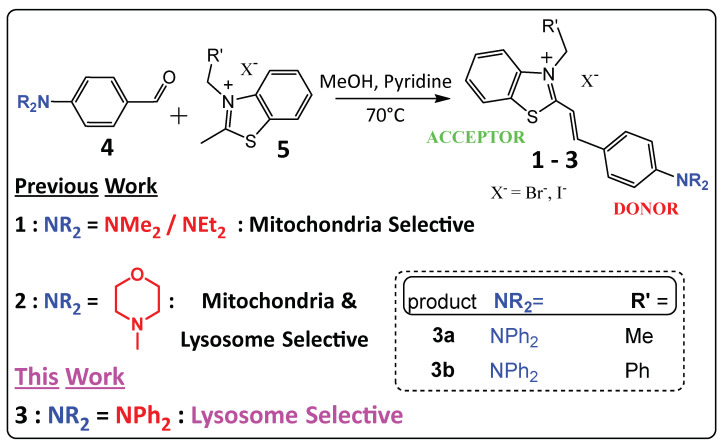
Schematic representation of the synthesis and the selectivity comparison in donor-π-acceptor probes **1**–**3**.

During our previous studies, we have reported the donor-π-accepter (D-π-A) based fluorescent probe **1** by attaching alkyl amine donor groups (i.e., R_2_N; where R = Me, Et) to the π-conjugated system of the dye (Scheme 1) [5,22]. When **1** was used in live-cell imaging experiments, they exhibited excellent selectivity towards intracellular mitochondria. However, when the donor group -NR_2_ was replaced by a morpholine group, probe **2** exhibited the ability to visualize both cellular mitochondria and lysosomes simultaneously in live-cell imaging experiments (Scheme 1) [31].

One intriguing question from our previous findings is why probes such as **1** or **2** with an organic amine group (-NR_2_ = -NMe_2_, pKa ≈ 5.15; and -NR_2_ = -NEt_2_, pKa ≈ 6.61) are not selective towards acidic cellular organelles such as lysosomes. This is in sharp contrast to many well-known fluorescent probes with basic functional groups (e.g., commercial LysoTracker^TM^ dyes) that have been used for visualizing acidic cellular organelles such as lysosomes (pH ≈ 4.6) [32,33,34]. In order to further investigate the effect of the donor (R_2_N-) group on the intra-cellular organelle selectivity, we decided to introduce a non-basic diphenyl amine (-NPh_2_) moiety as the donor group, which led to synthesis of probe **3**. On the basis of the previous study from **1**, one would expect a similar mitochondria selectivity in probe **3**. Surprisingly, probe **3** exhibited excellent selectivity towards cellular lysosomes (not mitochondria) during fluorescence confocal microscopy-based imaging studies. In this article, we discuss this unexpected selectivity switching in the cyanine-based D-π-A fluorescent dye system.

## 2. Materials and Methods

All chemicals for synthesis were purchased from Acros Organics and Sigma-Aldrich and used as received. Molecular biology-grade reagents for cell culture and fluorescent confocal microscopy experiments were purchased from Thermo Fisher. NMR characterization data were acquired on a Bruker 400 MHz NMR spectrometer. High-resolution mass spectrometric data were acquired using an ESI-TOF MS system (Waters, Milford, MA, USA). UV-vis studies were carried out in a Hewlett Packard-8453 diode array spectrophotometer at 25 °C. Fluorescence studies were conducted in a HORIBA Fluoromax-4 spectrofluorometer. Fluorescence confocal microscopy imaging was performed by a Nikon A1 confocal system with 100x oil objectives, a numerical aperture of 1.45, and a refractive index of 1.5. Throughout imaging, the temperature was maintained at 37 °C. Probes **3a**–**3b** were synthesized according to the previously reported procedure [35].

### 2.1. General Procedure for Synthesis

In a 25 mL round-bottom flask, 1.0 mmol. of 4-(4-Diphenylamino)benzaldehyde (**4**) was dissolved in 10 mL of methanol. Then, the appropriate 2-methylbenzothiazolium salt (**5**) (0.9 mmol) was added to the solution and stirred at room temperature for 5 min to result in a yellow-orange solution. Following the addition of pyridine (0.25 mL), the resulting solution was heated up to 70 °C for 12 h with stirring. Upon completion of the reaction (by TLC), the mixture was cooled down to room temperature, and ethyl acetate (20 mL) was added to the resulting dark brown solution. A red color solid product was precipitated in the bottom of the flask upon the addition of ethyl acetate. Then the solution was stirred for 10 min and allowed to settle down for another 10 min. The resulting solid products (**3a** or **3b**) were collected by vacuum filtration and further washed with ethyl acetate (3 × 20 mL) portions. Then, **3a** and **3b** were collected on the Buchner funnel as red color powders.

*(E)-2-(4-(diphenylamino)styryl)-3-ethylbenzo[d]thiazol-3-ium Iodide* (**3a**): Obtained as a dark brown solid with 77% isolated yield. ^1^H NMR (400 MHz, DMSO-*d6)* δ 8.39 (dd, *J* = 8.1, 1.2 Hz, 1H), 8.24 (d, *J* = 8.5 Hz, 1H), 8.16 (d, *J* = 15.5 Hz, 1H), 7.99–7.91 (m, 2H), 7.85 (ddd, *J* = 8.5, 7.3, 1.3 Hz, 1H), 7.83–7.71 (m, 2H), 7.49–7.40 (m, 4H), 7.30–7.21 (m, 2H), 7.25–7.17 (m, 4H), 6.96–6.88 (m, 2H), 4.91 (q, *J* = 7.2 Hz, 2H), 1.45 (t, *J* = 7.2 Hz, 3H). ^13^C NMR (101 MHz, DMSO) δ 171.28, 151.40, 149.17, 145.47, 140.87, 131.97, 130.00, 129.31, 128.01, 127.81, 126.17, 126.12, 125.44, 124.26, 119.01, 116.24, 109.49, 44.07, 14.03. HRMS (TOF MS ES^+^) found (*m/z*) for [M^+^] 433.1722. HRMS (calculated) found (*m/z*) for [M^+^] 433.1739.

*(E)-3-benzyl-2-(4-(diphenylamino)styryl)benzo[d]thiazol-3-ium Bromide* (**3b**): Obtained as a bright red solid with 75% isolated yield. ^1^H NMR (400 MHz, DMSO-*d6*) δ 8.42 (dd, *J* = 8.0, 1.4 Hz, 1H), 8.24 (d, *J* = 15.4 Hz, 1H), 8.17–8.10 (m, 1H), 7.95 (d, *J* = 15.4 Hz, 1H), 7.94–7.87 (m, 2H), 7.83–7.69 (m, 2H), 7.49–7.30 (m, 9H), 7.34–7.21 (m, 2H), 7.26–7.17 (m, 4H), 6.93–6.85 (m, 2H), 6.22 (s, 2H). ^13^C NMR (101 MHz, DMSO) δ 172.56, 151.64, 149.90, 145.34, 141.23, 133.98, 132.11, 130.02, 129.38, 129.11, 128.41, 128.08, 127.70, 126.85, 126.23, 125.99, 125.58, 124.38, 118.80, 116.57, 109.49, 51.04. HRMS (TOF MS ES^+^) found (*m/z*) for [M^+^] 495.1880. HRMS (calculated) found (*m/z*) for [M^+^] 495.1895.

### 2.2. Cell Culture, Staining, and Fluorescence Confocal Microscopy Imaging

Progenitor oligodendrocytes cells (MO3.13) were grown in DMEM (10% FBS and 1% penicillin/streptomycin added) were plated on MatTek 35 mm dish with a glass bottom at a density of 2 × 10^5^ cells/well. Plated cells were incubated overnight at 37 °C in a 5% CO_2_ environment. Following incubation, cells were washed with 1X PBS and stained with 200 nM (final concentration) probes (**3a** and **3b)** and appropriate commercial marker dyes in Gibco Live Cell Imaging Solution for 30 min. The initial cell staining experiments (probes **3a** and **3b** only) and co-localization experiments with commercial MitoTracker^TM^ Green FM (200 nM) or LysoTracker^TM^ Green DND-26 (70 nM) probes were conducted with a post-staining washing step with 1X PBS solution. Fluorescence confocal microscopy imaging was performed in Live Cell Imaging solution. All stock solutions of the fluorescent dyes for imaging experiments were prepared in 10 mM concentration in DMSO, and the % DMSO (*v*/*v*) in the imaging experiments was maintained below 0.5%.

Live-cell imaging studies were performed by a Nikon A1 confocal system with 100× oil objectives, a numerical aperture of 1.45, and a refractive index of 1.5. Imaging experiments were conducted at 37 °C temperature. Probe **3** was excited at 561 nm, and the emission was collected from 570 nm to 700 nm. MitoTracker^TM^ Green FM and LysoTracker^TM^ Green DND-26 dyes were excited at 488 laser, and the emission was collected from 495 nm to 550 nm range. The fluorescence confocal microscopy images were analyzed and processed by ImageJ (NIH) software. The Mander’s overlap coefficients (averaged) for co-localization analysis (with LysoTracker^TM^ Green DND-26 and MitoTracker^TM^ Green-FM dyes) were calculated by analyzing cell populations (n > 30) co-stained with probes **3a** and **3b** with standard co-localization analysis package equipped in the ImageJ (NIH) software.

For photostability comparison of the probe **3** with LysoTracker^TM^ Red DND-99, fluorescence confocal microscope was operated with following parameters: excitation laser—561 nm Laser line; power percentage 3.0 (2.1 mW); Digital zoom = 1; Pinhole = 1AU; Master Gain = 150; Digital offset = 0. MO3.13 cells were incubated with 100 nM dye concentration (all dyes) for 30 min and then continuously irradiated with 561 nm laser pulse. Fluorescence confocal microscopy images of the irradiated cells were acquired at 20 s intervals over a period of 4 min. ImageJ (NIH) software was used for analyzing images, and the average recovered fluorescence intensities (%) were plotted as a function of irradiation time.

## 3. Results and Discussion

Probe **3** was synthesized in good yields and characterized by NMR spectroscopy and high-resolution mass spectrometry as described in the materials and method section.

**Figure 1:**
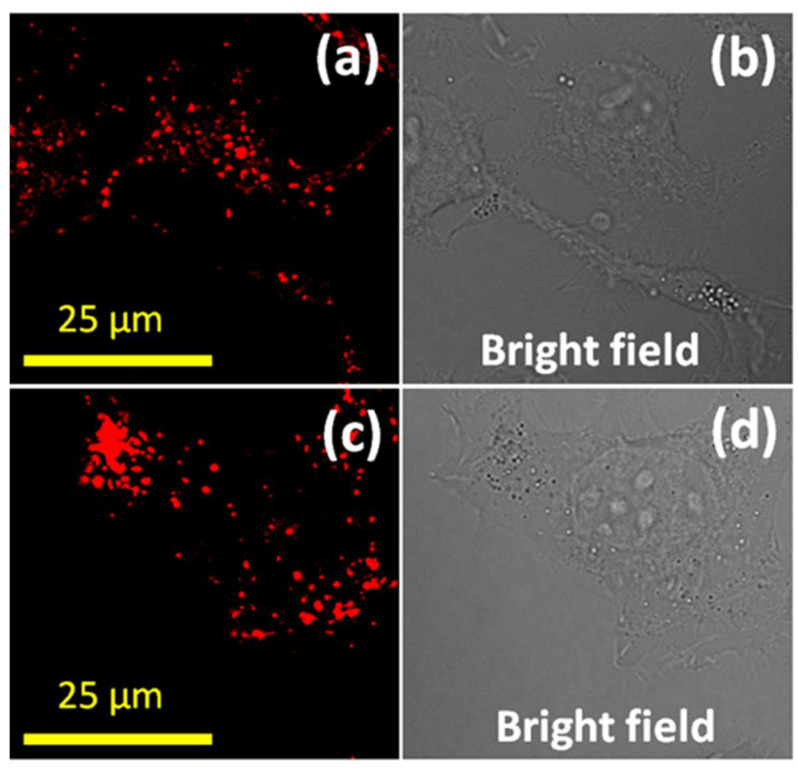
Fluorescence confocal microscopy images obtained from MO3.13 cells pre-incubated with probes **3a** (200 nM) and **3b** (200 nM) for 30 min. (**a**–**d**) represents probe **3a** emission within the cells (**a**), bright field (**b**), probe **3b** emission within the cells (**c**), and bright field (**d**). Probes **3a** and **3b** were excited with 561 nm laser line and the emission filter settings were set up to collect from 575 to750 nm range.

**Figure 2:**
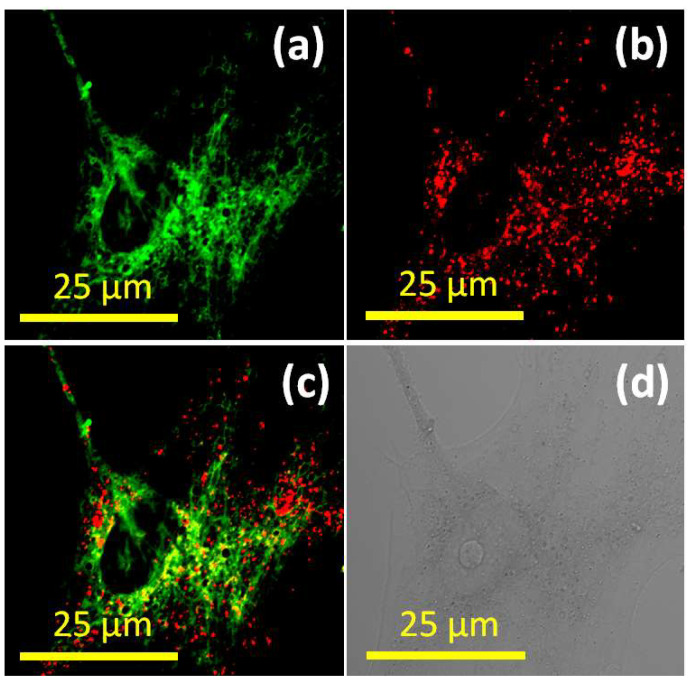
Fluorescence confocal microscopy images of the MO3.13 cells incubated with probe **3b** (200 nM) for 30 min in the presence of MitoTracker^TM^ Green FM (200 nM). Figures represent the staining of the MitoTracker^TM^ Green (**a**), the staining of **3b** (**b**), composite image (**c**), and the bright field (**d**). Probe **3b** was excited with 561 nm laser line and the MitoTracker^TM^ Green was excited with 488 nm laser line. The emission filter settings were set up to collect from 495 to 530 nm (MitoTracker^TM^ Green) and 575 to 750 nm (**3b**), respectively.

Although probes were nearly non-fluorescent in many organic and aqueous solvents, our recent work showed that probe **3** exhibits an intense red fluorescence turn ON upon binding to protein (i.e., albumin) [35]. By considering this interesting fluorescence turn ON phenomenon upon binding to protein (λ_em_ ≈ 630–640 nm; ϕfl ≈ 0.4), we hypothesized that such internalization of probe **3** in the intra-cellular environment (i.e., intra-cellular proteins or membranes) would also lead to a noticeable fluorescence turn ON. In order to test our hypothesis, Progenitor Oligodendrocytes (MO3.13) cells were pre-incubated with probes **3a** and **3b** (200 nM) for 30 min and analyzed by fluorescence confocal microscopy with 561 nm laser excitation. Surprisingly, MO3.13 cells stained with probes **3a** and **3b** produced a bright red non-uniform emission pattern, suggesting that probe **3** was likely localizing into a distinct organelle environment (Figure 1). Based on the observed pattern in Figure 1, probe **3** was not likely localizing in cellular mitochondria, as the imaging lacks the characteristic tubular-shaped staining pattern we observed for mitochondrial staining during our previous work [5,22]. This assumption was further verified by staining MO3.13 cells with **3a** and **3b** (200 nM) in the presence of MitoTracker^TM^ Green FM (200 nM). The fluorescence confocal microscope images (Figure 2) showed that probe **3** did not exhibit any noticeable mitochondria selectivity (calculated Mander’s overlap coefficients found to be 0.24–0.27), in sharp contrast to our previously reported probe **1** (Figure 2 and ESI Figure S2). The study was further repeated on Normal Human Lung Fibroblast (NHLF) cells for reproducibility and revealed a consistent pattern similar to MO3.13 cells (Figure 1 and ESI Figure S1).

**Figure 3:**
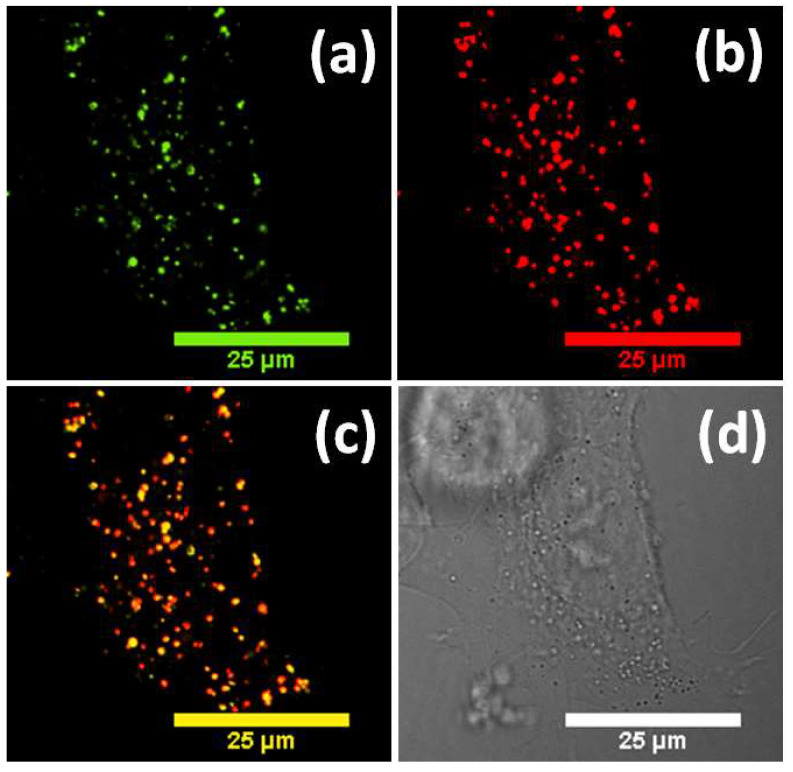
Fluorescence confocal microscope images of the MO3.13 cells incubated with probe **3b** (200 nM) for 30 min in the presence of LysoTracker^TM^ Green DND-26 (70 nM). Figures represent the staining of the LysoTracker^TM^ Green DND-26 (**a**), staining of probe **3b** (**b**), composite image (**c**), and the bright field (**d**). Probe **3b** was excited with 561 nm laser line and the LysoTracker^TM^ Green was excited with 488 nm laser line. The emission filter settings were set up to collect from 495 to 530 nm (LysoTracker^TM^ Green) and 575 to 750 nm (**3b**), respectively.

Interestingly, when MO3.13 cells were incubated with probes **3a** and **3b** (200 nM) in the presence of commercial LysoTracker^TM^ DND-26 (70 nM) and analyzed via fluorescence confocal microscopy, an excellent co-localization pattern was observed between probes **3a**–**3b** and LysoTracker^TM^ DND-26 (Figure 3). The calculated Mander’s overlap coefficients were found to be 0.89 for **3a** and 0.91 for **3b** in MO3.13 cells (Figure 3 and ESI Figures S3–S5). The results clearly pointed out that probe **3** is selective towards intracellular lysosomes. In order to further verify this finding, the imaging experiments were repeated by staining NHLF cells with probes **3a**–**3b** in the presence of LysoTracker^TM^ DND-26 (ESI Figures S3 and S4). As expected, the co-localization imaging patterns in NHLF cells also exhibited an excellent selectivity towards cellular lysosomes, with a calculated Mander’s overlap coefficient of 0.93 for (**3a**) and 0.94 (**3b**), respectively. Based on these studies, it was clear that the attachment of diphenylamine group (-NPh_2_) as the donor triggered a significant selectivity switching in the D-π-A dye system from mitochondria to lysosome. In other words, the donor group (-NPh_2_) in the D-π-A system had a large impact on the probe’s selectivity toward the subcellular organelles. Based on these experimental data, switching from an alkyl amine donor group (NR_2_: NMe_2_ or NEt_2_) to an aromatic amine donor group (-NPh_2_) was the key parameter for observed organelle selectivity switching in the probe.

**Figure 4:**
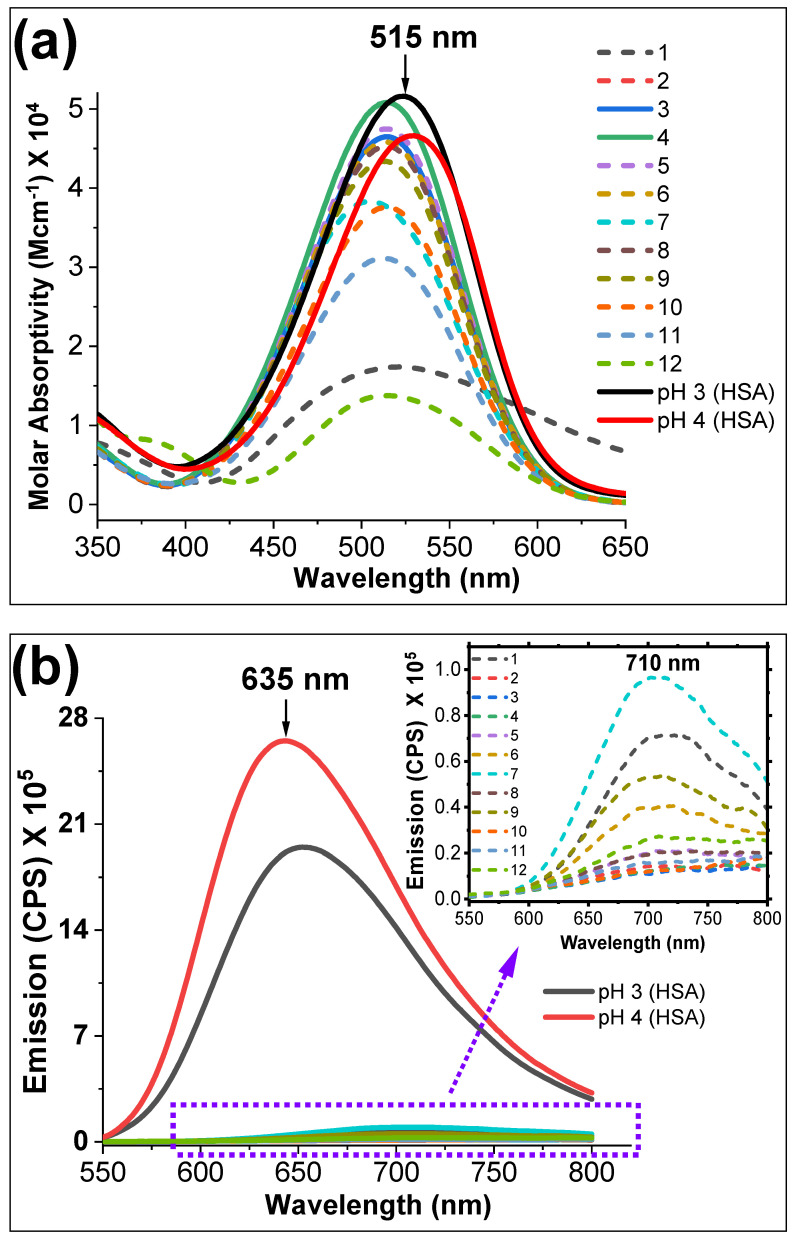
Absorption (**a**) and fluorescence (**b**) spectra were recorded for **3b** (1 × 10^−5^ M) in different aqueous acidic environments. At pH, 10 µL of 10% HSA solution was introduced into the acidic solutions of probe **3b**. The probe was excited at 510 nm and the emission was recorded from 550 nm to 800 nm.

Probe **3** gave extremely weak near-infrared (NIR) emission (λ_em_ > 700 nm and φfl < 0.005) in many different solvents (Table 1 and ESI Figure S7). However, probes exhibited a bright red emission (λ_em_ ≈ 630–640 nm) during live-cell imaging with extremely lower concentrations such as 200 nM (Figure 1 and Figure 2 and ESI Figures S3–S5). The observed bright red emission was consistent with our recently reported albumin-induced fluorescence turn ON in probes **3a** and **3b** [35]. In order to shed some light on understanding the behavior of probe **3** in cellular lysosomes to turn on a bright red emission, we first decided to examine the optical properties of probes **3a** and **3b** in different aqueous acidic environments (pH 1–12) (Figure 4 and ESI Figures S9–S13). Probes exhibited a very weak NIR emission (λ_em_ ≈ 700–730 nm) over a wide pH range (Figure 4 and ESI Figure S10). Interestingly, probes exhibited a higher molar absorptivity in the pH range 3–5, which mimicked typical lysosomal lumen acidity (Figure 4a and ESI Figure S11). Therefore, one intriguing question to be raised is how these probes exhibit a bright red fluorescence upon being internalized into cellular lysosomes. From our previously reported work, probes internalized into hydrophobic lysosomal membrane environments exhibited two consistent characteristics. (1) Their bright emission in organic solvents and (2) significant fluorescence quantum yield difference in organic vs. aqueous solvents, which enables us to perform “wash-free” staining with the probes [2,5,12]. In sharp contrast, probe **3** did not exhibit either of these characteristics, which triggered us to think that the internalization location of probe **3** was likely not to be the lysosomal membrane [12]. Based on spectroscopic studies and fluorescence microscopy imaging results, we hypothesized that probes **3a**–**3b** likely populated into the acidic lysosomal lumen and stabilized/shielded by internalizing into hydrophobic environments such as lysosomal lumen proteins. In order to test our hypothesis, we designed an aqueous solution-based model to study the probe’s emission in an acidic environment with and without introducing a soluble protein (i.e., Albumin). Initially, the emissions of probes **3a** and **3b** were recorded in aqueous solutions under different pH conditions (Figure 4 and ESI Figure S13). In summary, probes exhibited a very weak NIR emission (λ_em_ ≈ 700–730 nm) in all pH conditions (Figure 4 and ESI Figure S13). Then, aqueous 10% Human Serum Albumin (HSA) was introduced into solutions that mimic lysosomal pH ranges (pH 3–5) (Figure 4 and ESI Figure S13). Interestingly, a bright red emission (λ_em_ ≈ 630–640 nm) was observed in the aqueous acidic solutions upon the addition of HSA). In addition, the spectrometric titration of probes **3a**–**3b** with HSA at pH4 exhibited a significant fluorescence turn on at λ_em_ ≈ 620–640 with increasing protein concentration (ESI Figure S14). The observed results indicated strong evidence of possible internalization of probe **3** into the hydrophobic environments of the protein (i.e., binding pockets) from the acidic aqueous environment to turn ON a bright red emission (λ_em_ ≈ 620–640 nm; φ_fl_ ≈ 0.4) [35]. Also, unnoticeable changes in the absorption profile of probes **3a** and **3b** in the presence/absence of the HSA provided strong evidence to indicate the distribution of probe **3** as a suspension in solution rather than a precipitate/aggregate (i.e., lower absorption). Also, an *unchanged* absorption spectra pattern (Figure 4a and Figure S13 further indicated that no structural alterations occurred upon the addition of the protein into acidic environments. The resulting bright red emission (λ_em_ ≈ 620–640 nm; φ_fl_ ≈ 0.4) with a noticeable hypsochromic shift (∆λ ≈ 75 nm) (Figure 4b) in acidic environments was similar to our previously reported findings [35]. Based on these experimental findings, we proposed that probe **3** may likely disperse in acidic lysosomal environments (i.e., lumen) while internalizing into hydrophobic environments in the lysosomal lumen components (i.e., lumen proteins) to turn on bright red fluorescence.

**Figure 5:**
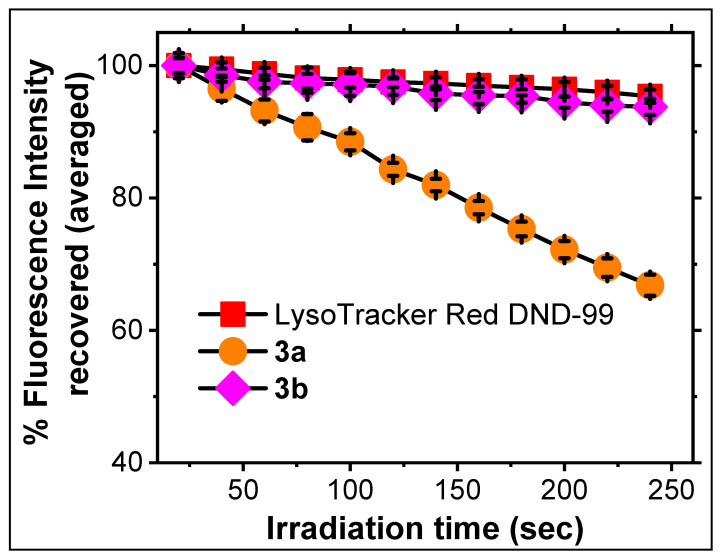
The plot of recovered fluorescence intensity versus irradiation time calculated for probes **3a**, **3b** and LysoTracker^TM^ Red DND-99 (100 nM) in MO3.13 cells upon continuous irradiation (Laser power percentage 3.0; Digital zoom = 1; Pinhole = 1AU; Master Gain = 150; Digital offset = 0).

In order to further evaluate the reliability of probe **3** for live-cell imaging applications, we decided to investigate the photostability of **3** by exposing it to continuous laser irradiation in the fluorescence microscope. MO3.13 cells stained with probes **3a** and **3b** (100 nM concentration) were continuously irradiated with 561 nm laser line (Laser power percentage 3.0; Digital zoom = 1; Pinhole = 1AU; Master Gain = 150; Digital offset = 0) for a period of 4 min while obtaining image frames at 20 s intervals. The recovered percentage fluorescence intensity after irradiation was plotted as a function of the time for each irradiation frame (Figure 5). As a reference for comparison, MO3.13 cells, stained with commercial LysoTracker^TM^ Red DND-99 (100 nM), were irradiated under identical microscope settings (Figure 5). Based on the recovered fluorescence intensity, both probe **3b** and LysoTracker^TM^ Red DND-99 exhibited exceptional photostability. However, in sharp contrast, probe **3a** was highly susceptible to photobleaching due to continuous irradiation. These results indicated that the attachment of benzyl substituent into the nitrogen atom of the benzothaiazolium moiety (i.e., **3b**) plays a key role in increased photostability in comparison to the ethyl substituent (i.e., **3a**) [12]. Therefore, probe **3b** sustains a highly useful architectural design for developing novel non-alkalinizing D-π-A-based fluorescent dyes for lysosome imaging in live cells.

## 4. Conclusions

In conclusion, a new D-π-A cyanine dye **3** with a diphenylamine (-NPh_2_) donor group has been successfully used in live-cell imaging applications by fluorescence confocal microscopy. As a result of incorporating a diphenylamine donor, the mitochondria selectivity of this D-π-A cyanine dye was completely altered, where an exceptional lysosome selectivity was observed for probes **3a** and **3b**. Based on our fundamental studies, probe **3** was likely internalized into the proteins present in the lumen of the cellular lysosome rather than to the lysosomal membrane. In addition, probe **3** skeleton can be further tailored for improved photostability by incorporating benzyl substituent in the cyanine core. Probe **3b** in the presence of benzyl substituent in the thiazolium moiety exhibited relatively high photostability and improved lysosome specificity in comparison to **3a**. Probe **3** will be an interesting candidate for developing highly biocompatible lysosome selective fluorescent probes for live-cell imaging applications.

## Figures and Tables

**Table 1:** Spectroscopic properties of **3a** and **3b**.

Solvent	3a			3b		
	λ_abs_ (nm)	λ_em_ (nm)	ϕ_fl_	λ_abs_ (nm)	λ_em_ (nm)	ϕ_fl_
DCM	549	734	0.003	558	747	0.001
ACN	507	720	0.0005	520	750	0.0003
DMSO	506	728	0.002	519	745	0.002
EtOH	518	708	0.003	532	711	0.002
Water	501	720	0.0001	517	731	0.0001

## Supplementary Materials

The following supporting information can be downloaded at: https://www.mdpi.com/article/10.3390/bios12070504/s1, Figures S1–S5: Cell imaging and analysis; Figures S6–S13: Study of photophysical properties; Figure S14: Characterization data.
