# Supplementary material for: The Unexpected Selectivity Switching from Mitochondria to Lysosome in a D-π-A Cyanine Dye
Source: Biosensors (Basel). 2022 Jul 10;12(7):504. doi: 10.3390/bios12070504 (PMC9313378; doi:10.3390/bios12070504)
Supplement: Supplementary file 1 [file biosensors-12-00504-s001.zip › biosensors-1800621-supplementary.pdf]

The Donor-Induced Unexpected Selectivity Switching from Mitochondria to Lysosome in a Cyanine Dye

Supporting Information

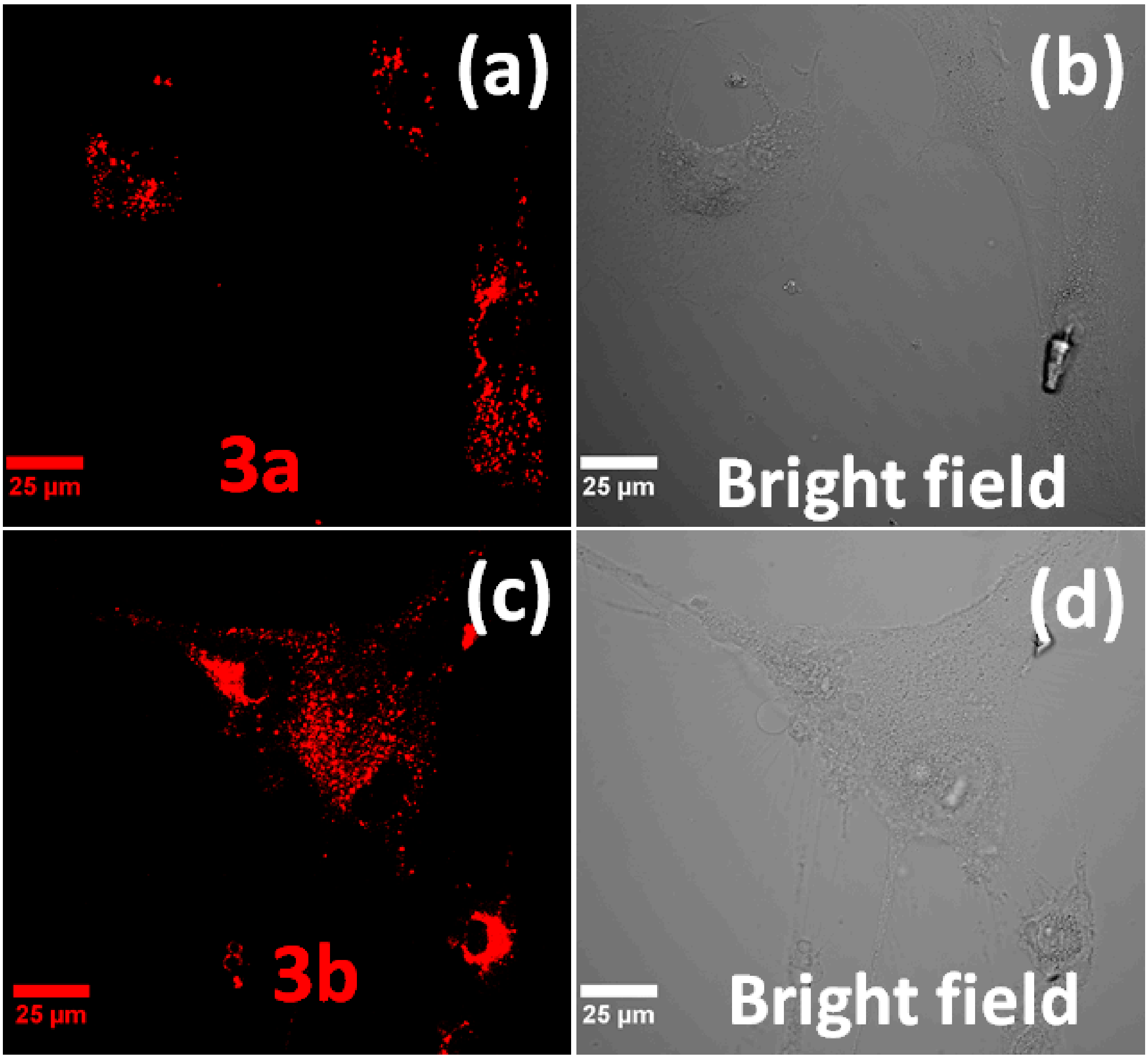

Figure S1 Fluorescence confocal microscopy images obtained from NHLF cells pre-incubated with probe **3a** (200 nM) and **3b** (200 nM) for 30 minutes. Images a-d represents, probe **3a** emission within the cells (a), bright field (b), probe **3b** emission within the cells (c) and bright field (d). Probe **3a** and **3b** were excited with 561 nm laser line and the emission filter setting were set up to collect from 575 – 750 nm range.

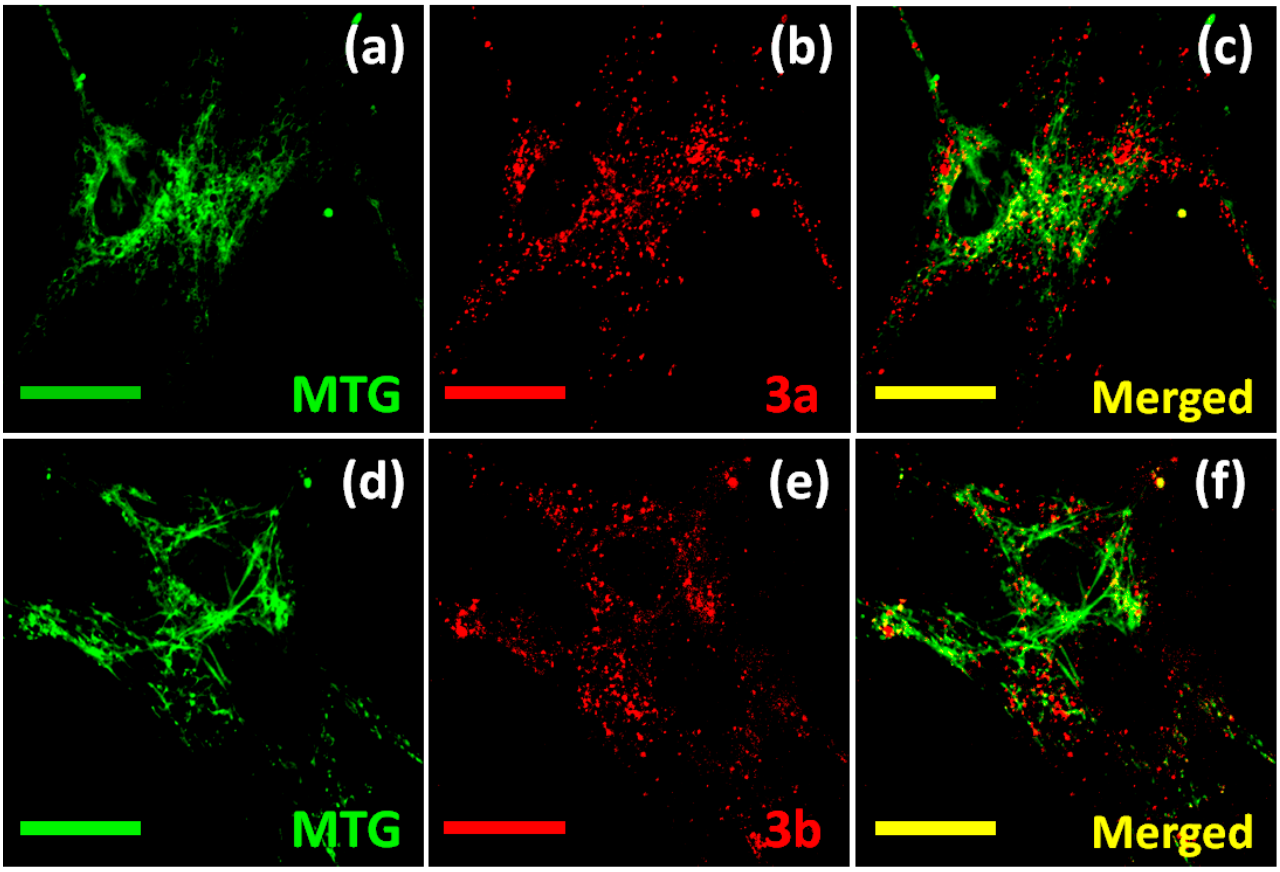

Figure S2 Fluorescence confocal microscopy images of the MO3.13 cells incubated with probe **3a** (200 nM) and **3b** (200 nM) for 30 minutes in the presence of MitoTracker™ Green FM (200 nM). Figures a and d represents the staining of the MitoTracker™ Green, figures b and e represents the staining of the probe **3a** and **3b** respectively. Figures c and f represents the composite images. Probe **3a** and **3b** were excited with 561 nm laser line and the MitoTracker™ Green was excited with 488 nm laser line. Calculate Mander's overlap coefficients found to be 0.27 (**3a**) and 0.24 (**3b**). The emission filter settings were set up to collect from 495-530 nm (MitoTracker™ Green) and 575 – 750 nm (**3**) respectively.

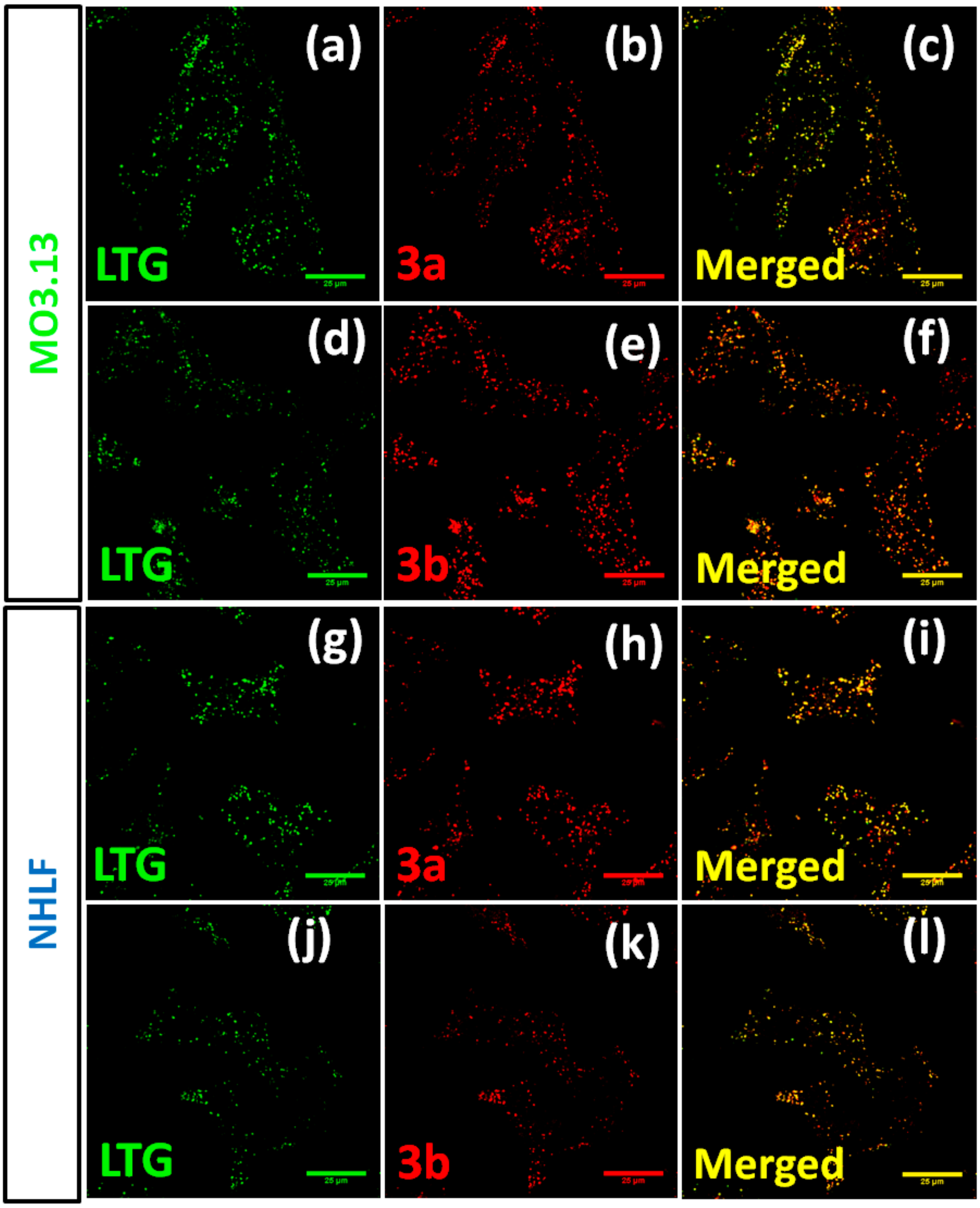

Figure S3 Fluorescence confocal microscopy images of the MO3.13 cells (figures a – f) and NHLF cells (figures g – l) incubated with probe **3a** (200 nM) and **3b** (200 nM) for 30 minutes in the presence of LysoTracker^{TM} Green DND-26 (70 nM). Figures a,d,g and j represents the staining of the LysoTracker^{TM} Green, figures b and h represents the staining of the probe **3a**, figures e and k represents the staining of probe **3b** and figures c, f, I, and l represents the relevant composite images. Probe **3a** and **3b** were excited with 561 nm laser line and the LysoTracker^{TM} Green was excited with 488 nm laser line. Calculate Mander's overlap coefficient for **3a** found to be 0.91 (MO3.13) and 0.94 (NHLF). Calculated Mander's overlap coefficient for **3b** found to be 0.89 (MO3.13) and 0.93 (NHLF). The emission filter settings were set up to collect from 495-530 nm (LysoTracker^{TM} Green) and 575 – 750 nm (**3**) respectively.

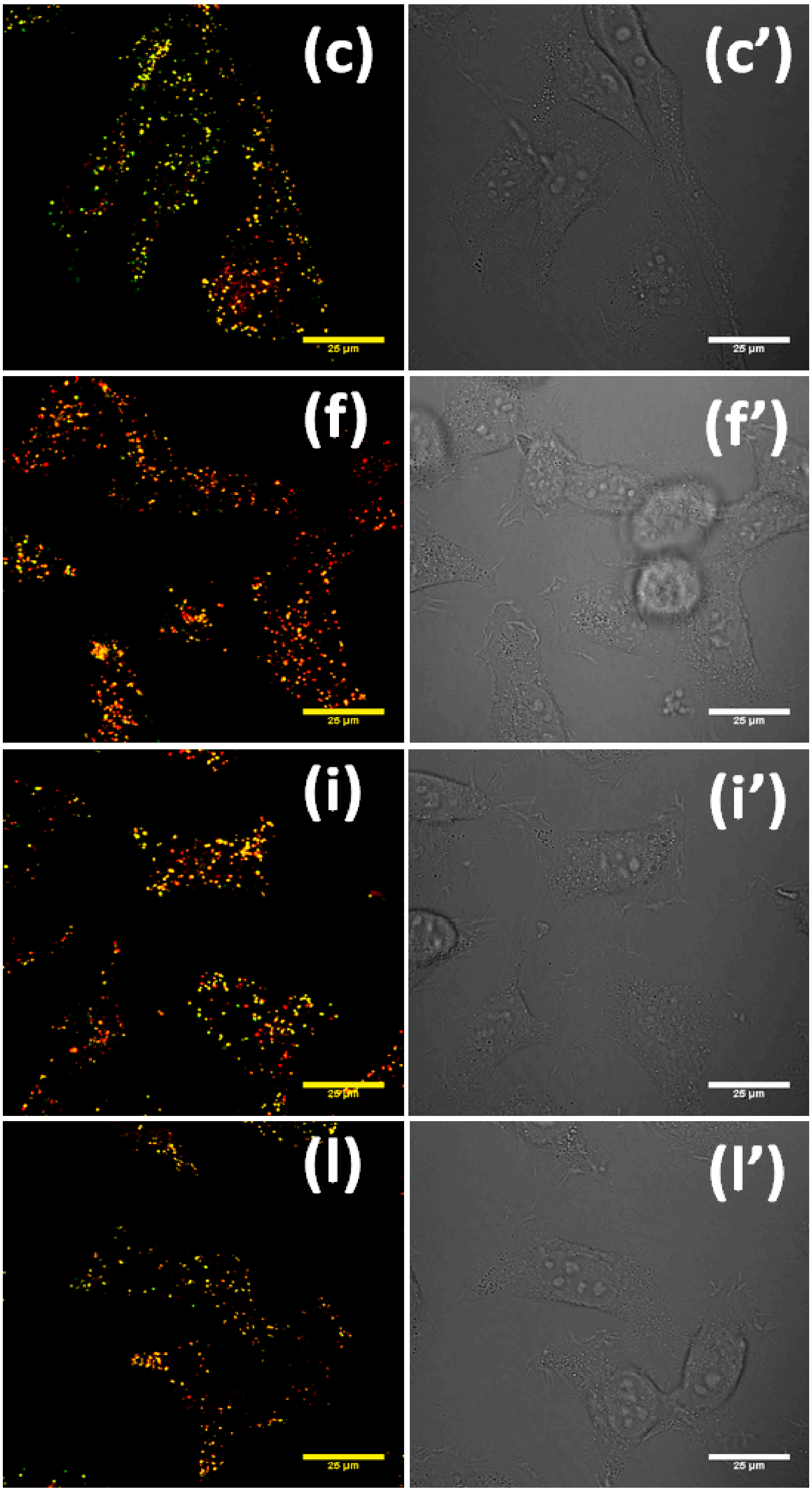

Figure S4 Represents the corresponding bright field images (c', f', i' and l') for each composite image presented in figure **S3** (c, f, i and l).

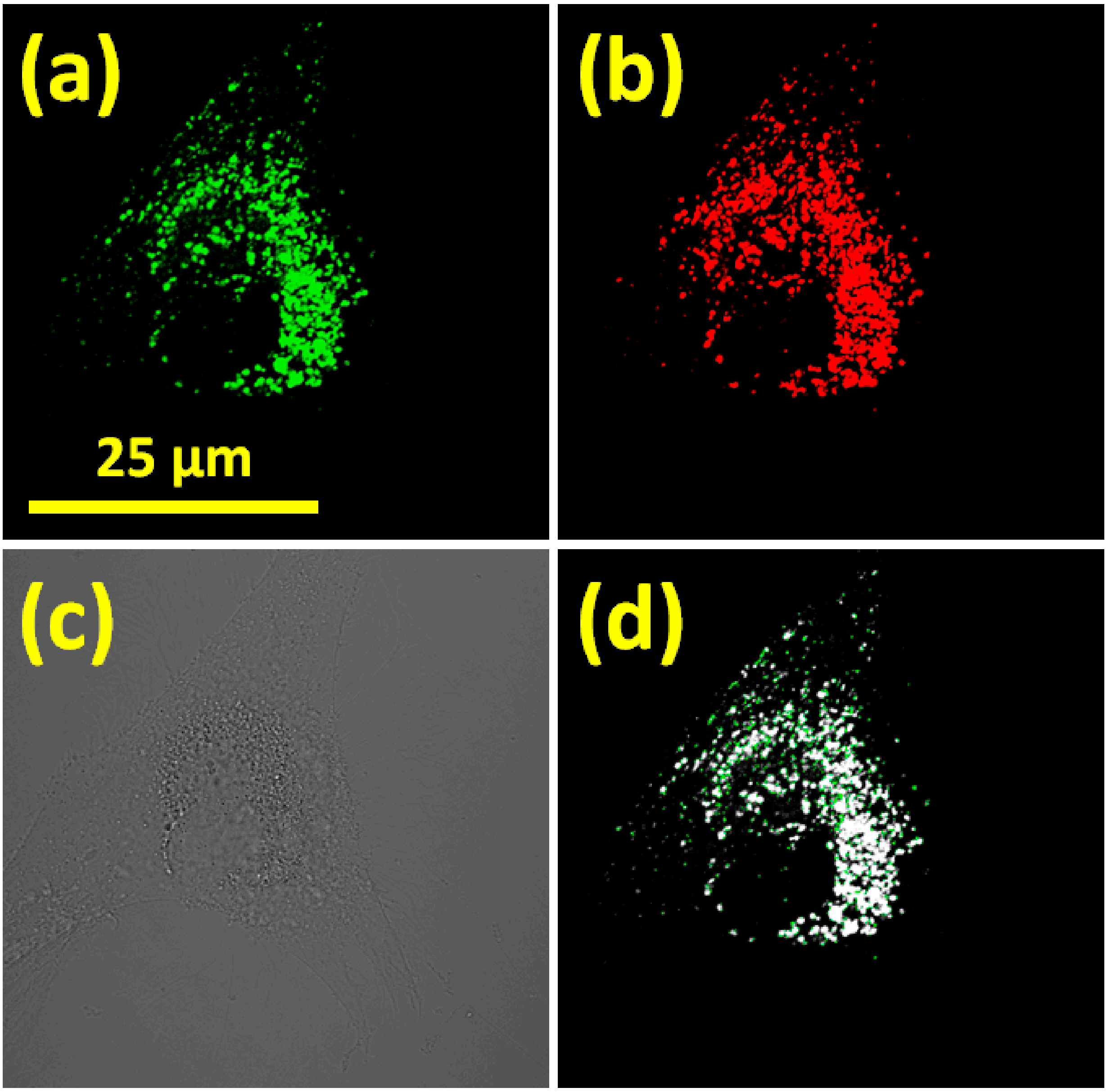

Figure S5.1 Colocalization analysis for the fluorescence confocal microscopy images obtained for probe **3a** (200 nM) in MO3.13 cells with LysoTracker^{TM} Green DND-26 (70 nM). Images from a to c represents, the staining pattern of the LysoTracker^{TM} Green DND-26 (a), staining pattern of the probe **3a** (b) and the bright field image (c). Image d represents the digital co-localization analysis performed for probe **3a** and LysoTracker^{TM} Green DND-26 by ImageJ (NIH) software. The grey/white area represents the perfect overlap region. The calculated Mander's overlap coefficient for the analysis is 0.89.

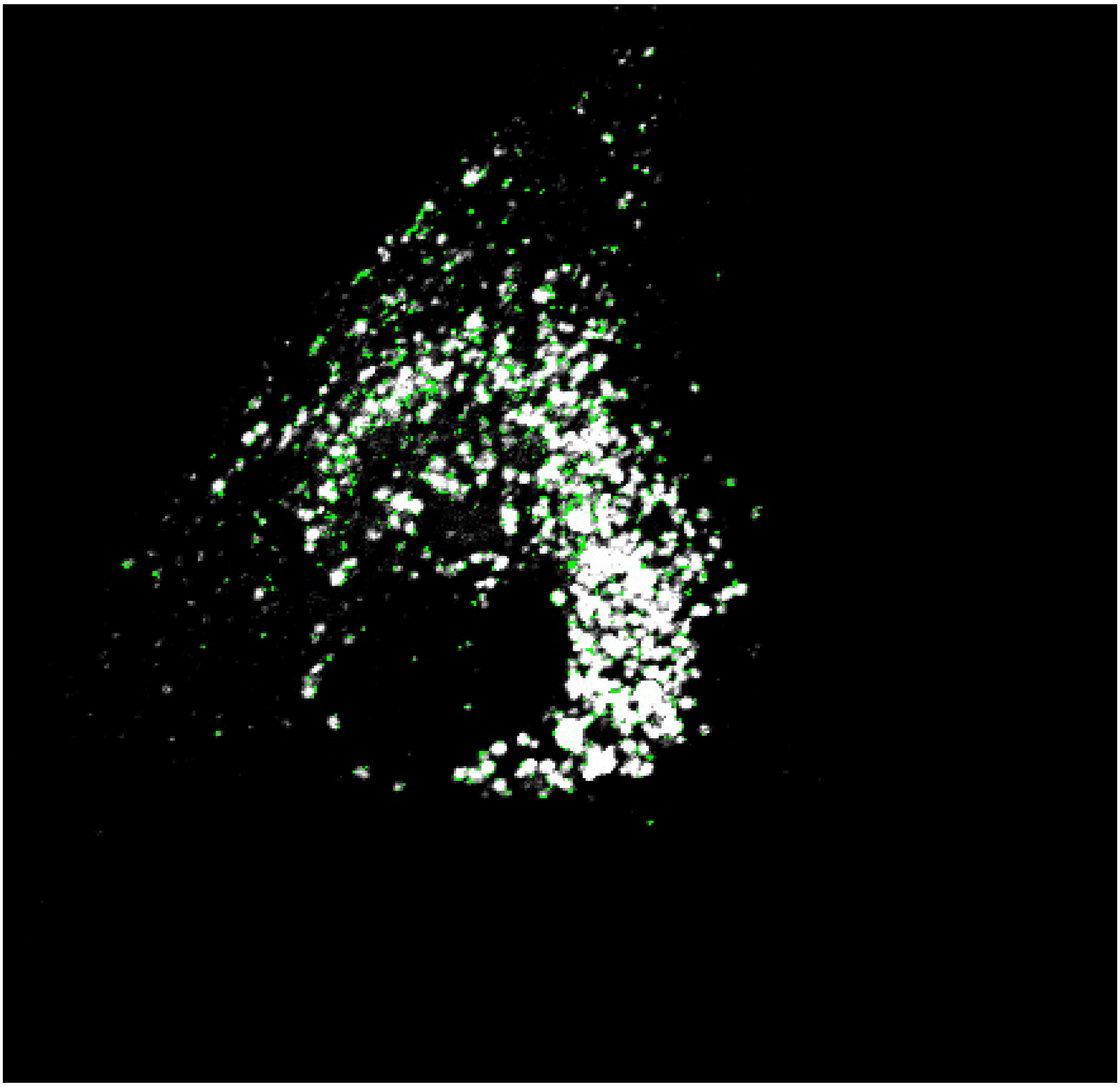

Figure S5.2 Represents the zoomed-in version of the figure S5.1d for digital co-localization analysis performed for probe **3a** and LysoTracker^{TM} Green DND-26 by ImageJ (NIH) software. The grey/white area represents the perfect overlap region. The calculated Mander's overlap coefficient for the analysis is 0.89.

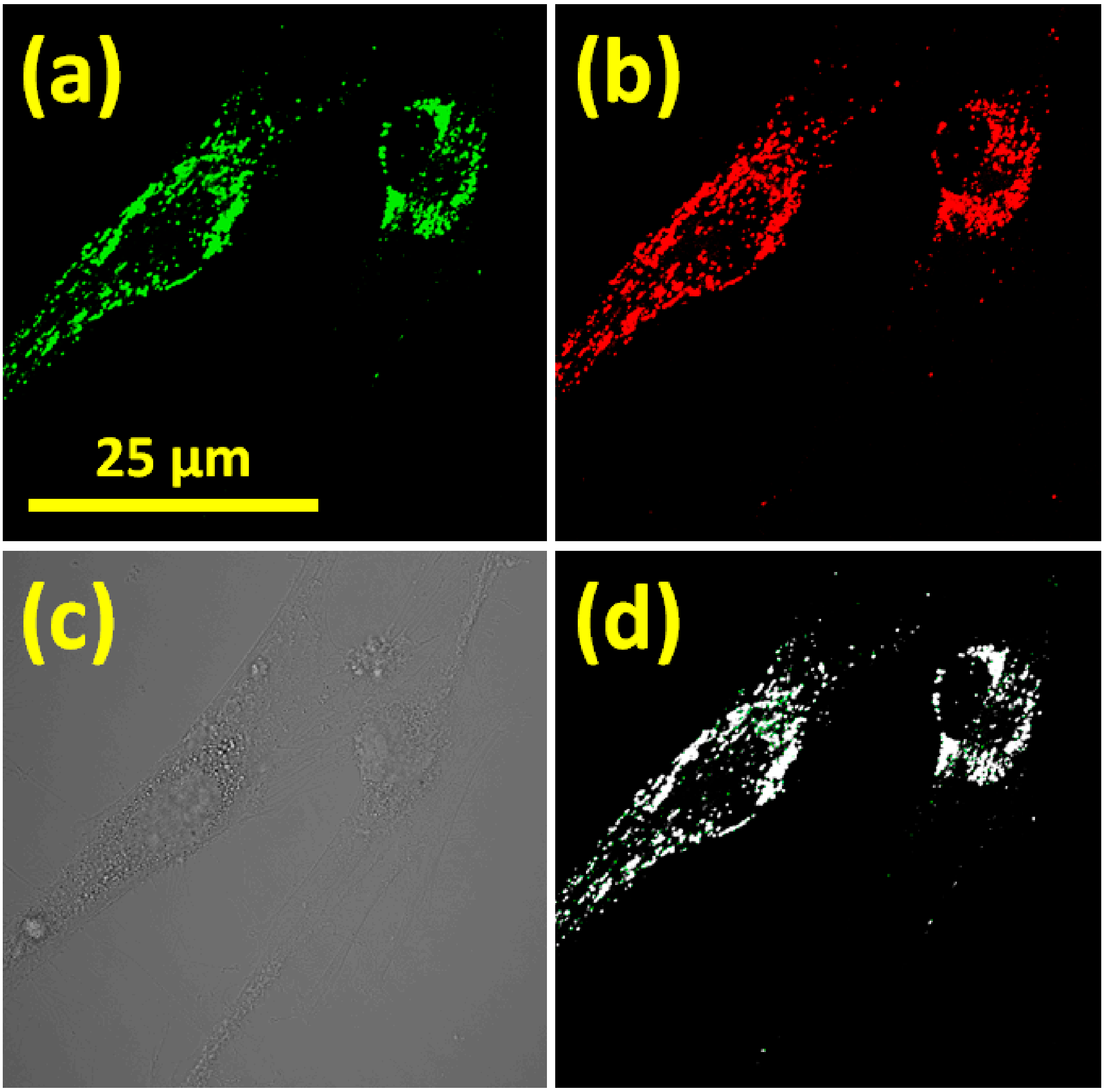

Figure S5.3 Colocalization analysis for the fluorescence confocal microscopy images obtained for probe **3b** (200 nM) in MO3.13 cells with LysoTracker^{TM} Green DND-26 (70 nM). Images from a to c represents, the staining pattern of the LysoTracker^{TM} Green DND-26 (a), staining pattern of the probe **3b** (b) and the bright field image (c). Image d represents the digital co-localization analysis performed for probe **3b** and LysoTracker^{TM} Green DND-26 by ImageJ (NIH) software. The grey/white area represents the perfect overlap region. The calculated Mander's overlap coefficient for the analysis is 0.91.

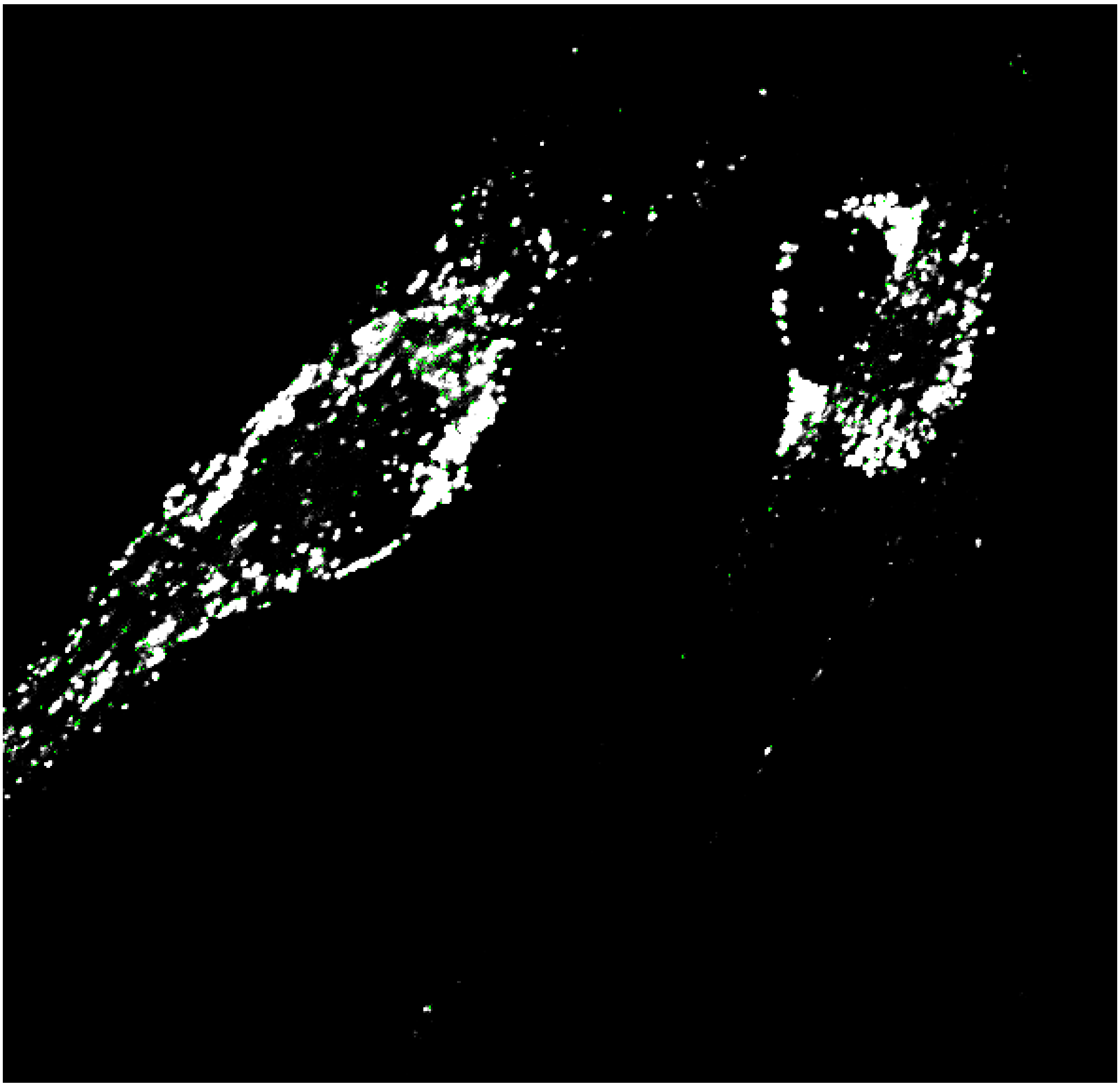

Figure S5.4 Represents the zoomed-in version of the figure S5.3d for digital co-localization analysis performed for probe **3b** and LysoTracker™ Green DND-26 by ImageJ (NIH) software. The grey/white area represents the perfect overlap region. The calculated Mander's overlap coefficient for the analysis is 0.91.

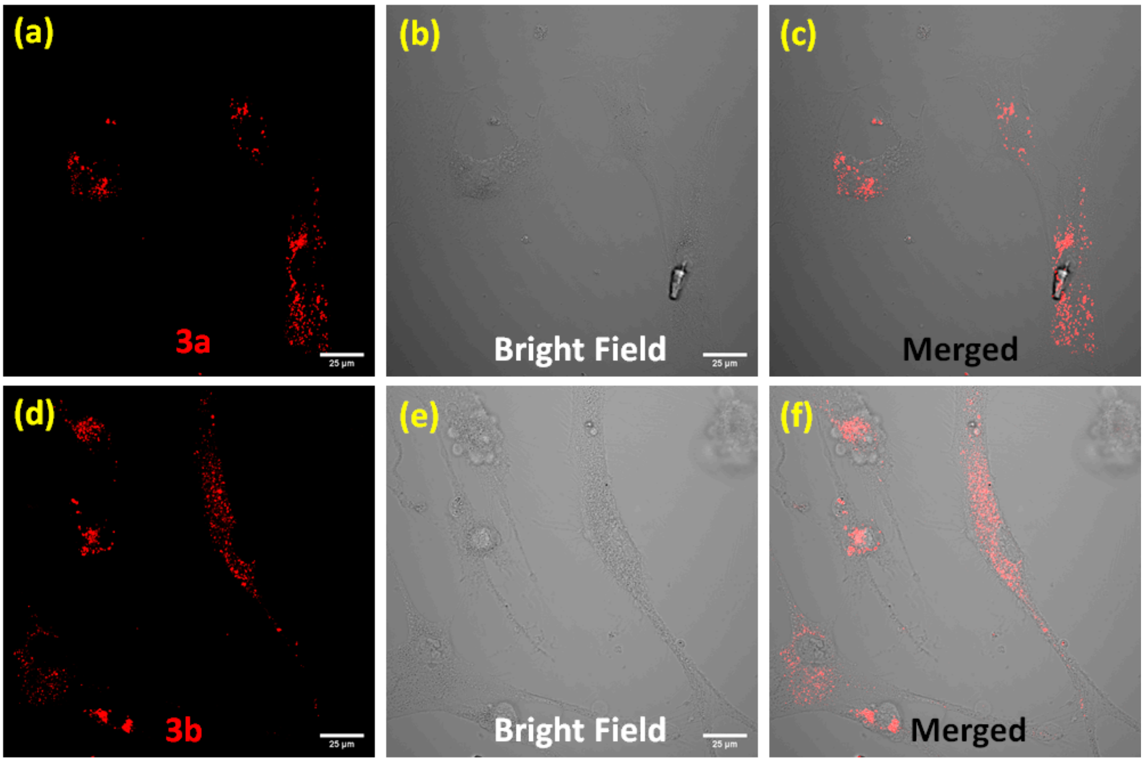

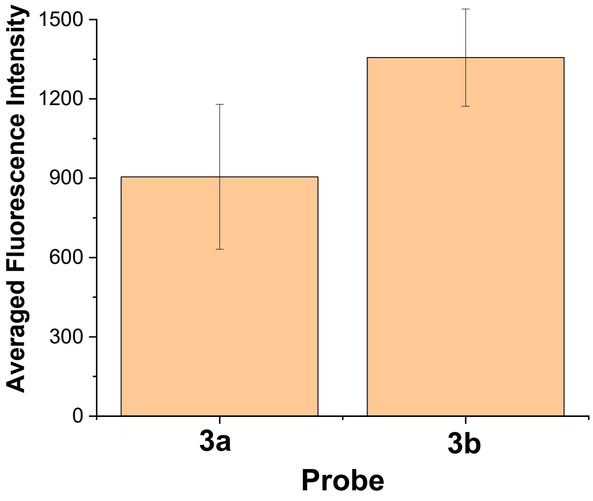

Figure S5.5 Representative fluorescence confocal microcopy images of MO3.13 cells incubated with 200nM probe **3a** (a-c) and **3b** (d-f). The merged images c and f were processed by combining the probe **3a**-**3b** images with their respective bright filed images to represent the cellular boundary.

The bar-chart represents the averages fluorescence intensity calculated from the MO3.13 cells stained with 200nM probe **3a** and **3b**. The averaged fluorescence intensity was calculated by ImageJ(NIH) software with two set of replicates (2 x n; where n=15)

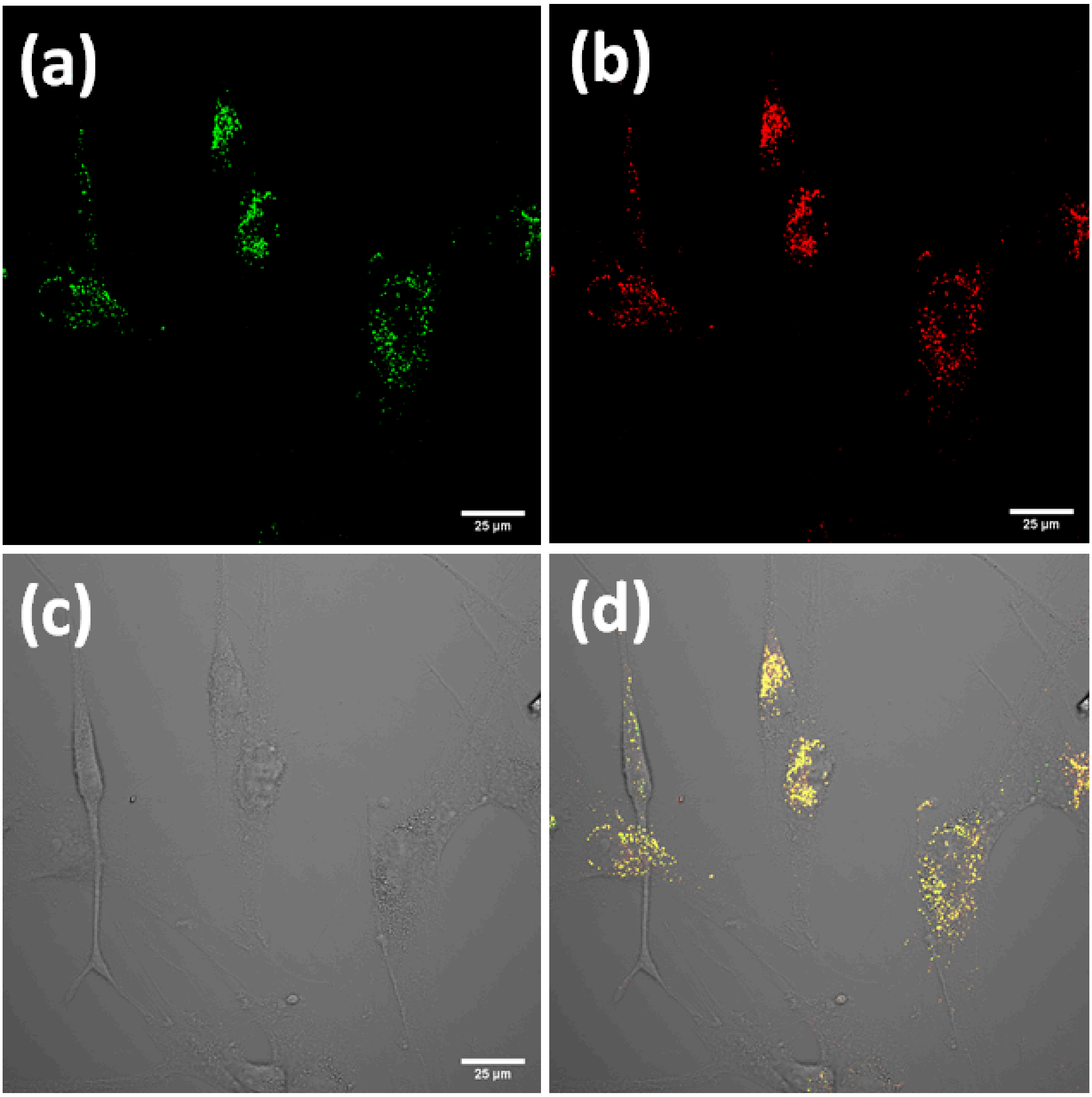

Figure S5.6 Representative fluorescence confocal microscope images of the MO3.13 cells incubated with probe **3b** (200 nM) for 30 minutes in the presence of LysoTracker^{TM} Green DND-26 (70 nM). Probe **3b** was excited with 561 nm laser line and the LysoTracker^{TM} Green was excited with 488 nm laser line. The emission filter settings were set up to collect from 495-530 nm (LysoTracker^{TM} Green) and 575 – 750 nm (**3b**) respectively.

Figures represents the staining of the LysoTracker^{TM} Green DND-26 (a), staining of the probe **3b** (b), bright filed image (c) and composite image to represent the cellular boundaries(d).

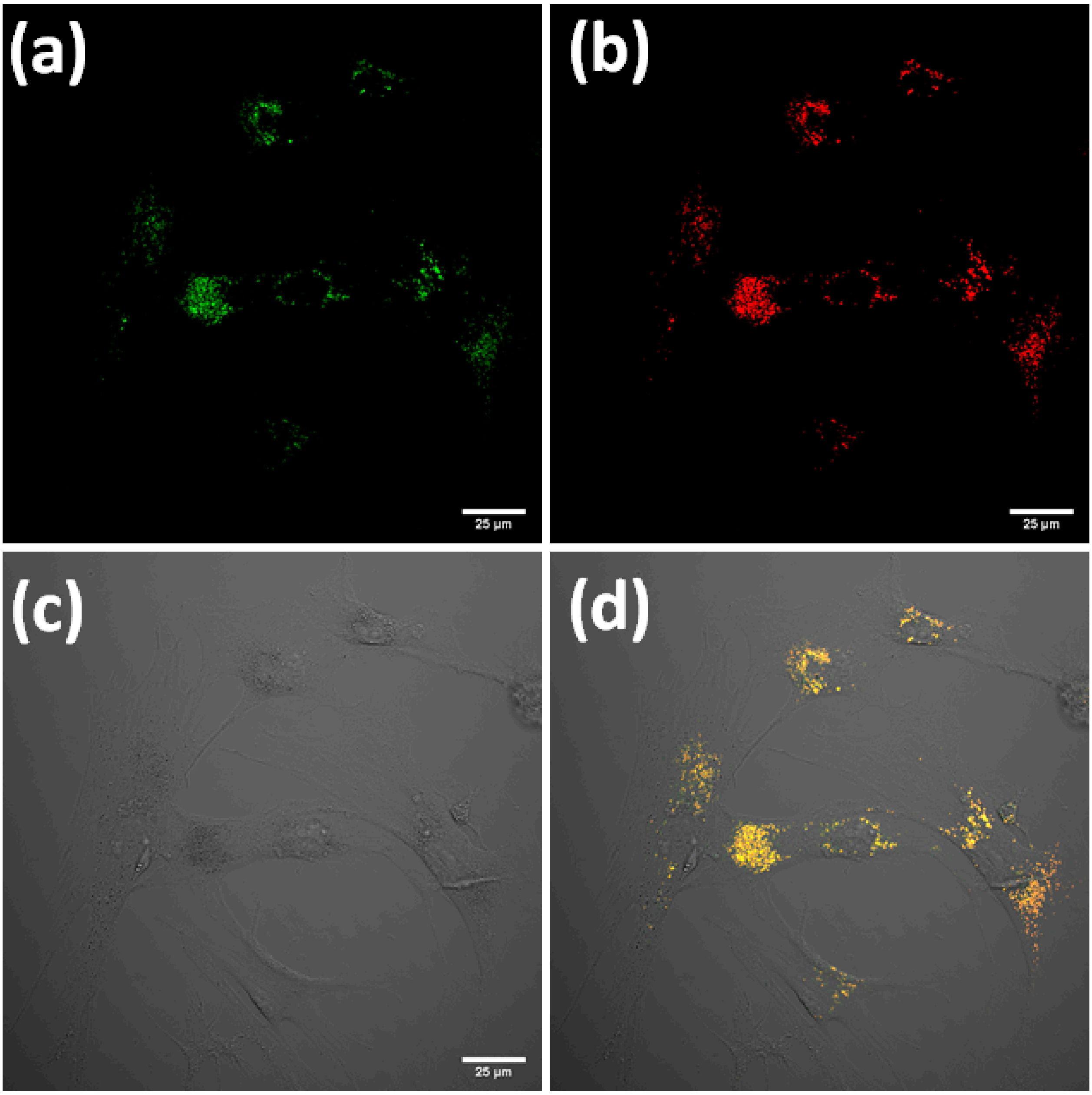

Figure S5.7 Representative fluorescence confocal microscope images of the MO3.13 cells incubated with probe **3a** (200 nM) for 30 minutes in the presence of LysoTracker™ Green DND-26 (70 nM). Probe **3a** was excited with 561 nm laser line and the LysoTracker™ Green was excited with 488 nm laser line. The emission filter settings were set up to collect from 495-530 nm (LysoTracker™ Green) and 575 – 750 nm (**3a**) respectively.

Figures represents the staining of the LysoTracker™ Green DND-26 (a), staining of the probe **3a** (b), bright filed image (c) and composite image to represent the cellular boundaries(d).

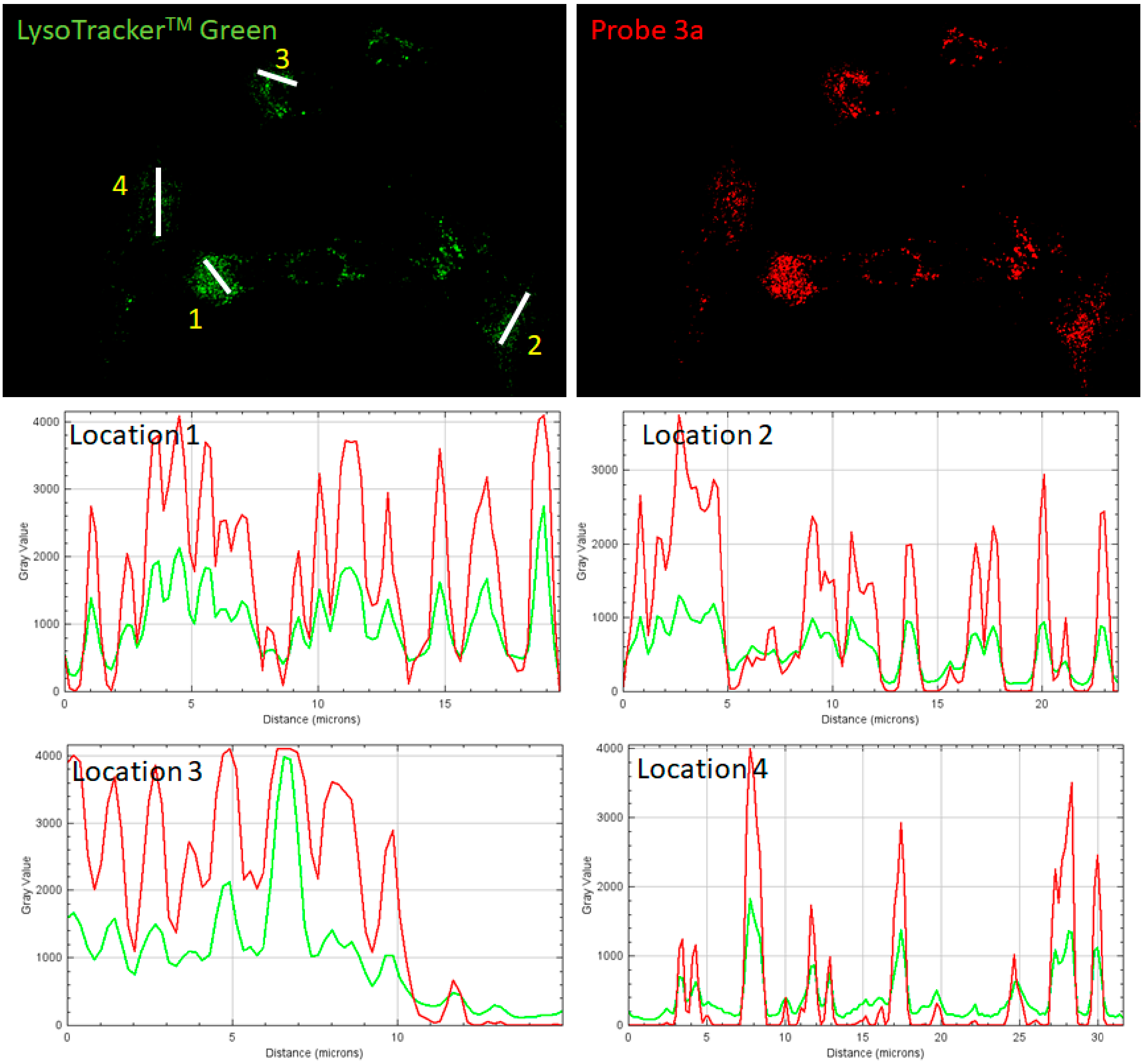

Figure S5.8 Representative fluorescence confocal microscope images of the MO3.13 cells incubated with probe **3a** (200 nM) for 30 minutes in the presence of LysoTracker™ Green DND-26 (70 nM). Fluorescence intensity vs distance plots were obtained for selected regions (locations 1-4) to represent the co-localization pattern.

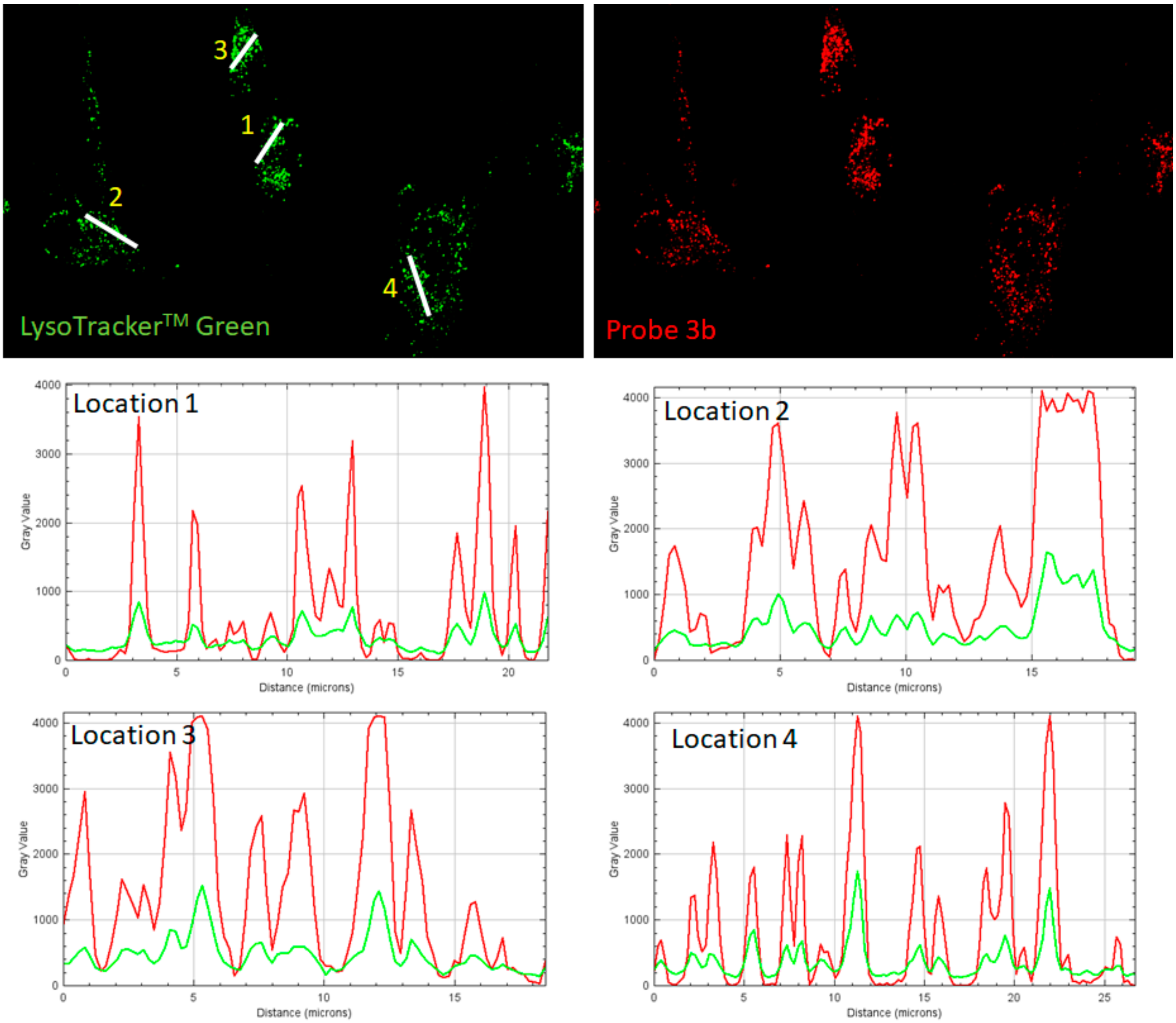

Figure S5.9 Representative fluorescence confocal microscope images of the MO3.13 cells incubated with probe **3b** (200 nM) for 30 minutes in the presence of LysoTracker^{TM} Green DND-26 (70 nM). Fluorescence intensity vs distance plots were obtained for selected regions (locations 1-4) to represent the co-localization pattern.

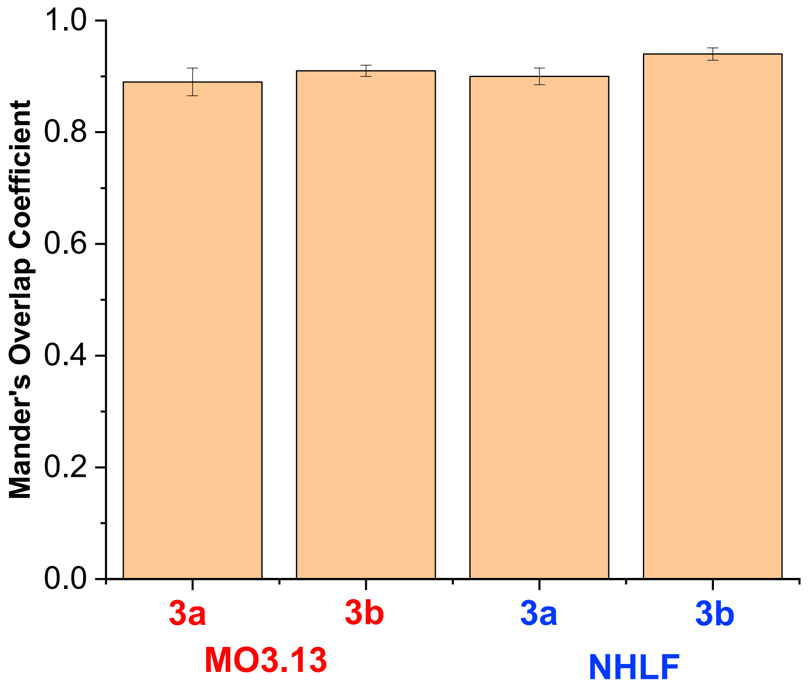

Figure S5.10 The averaged Mander's overlap coefficient calculated for probes **3a** and **3b** in the presence of LysoTracker^{TM} Green DND-26 in MO3.13 and NHLF cells. The averaged Mander's overlap coefficient was calculated by taking 30 individual cell samples for each entry.

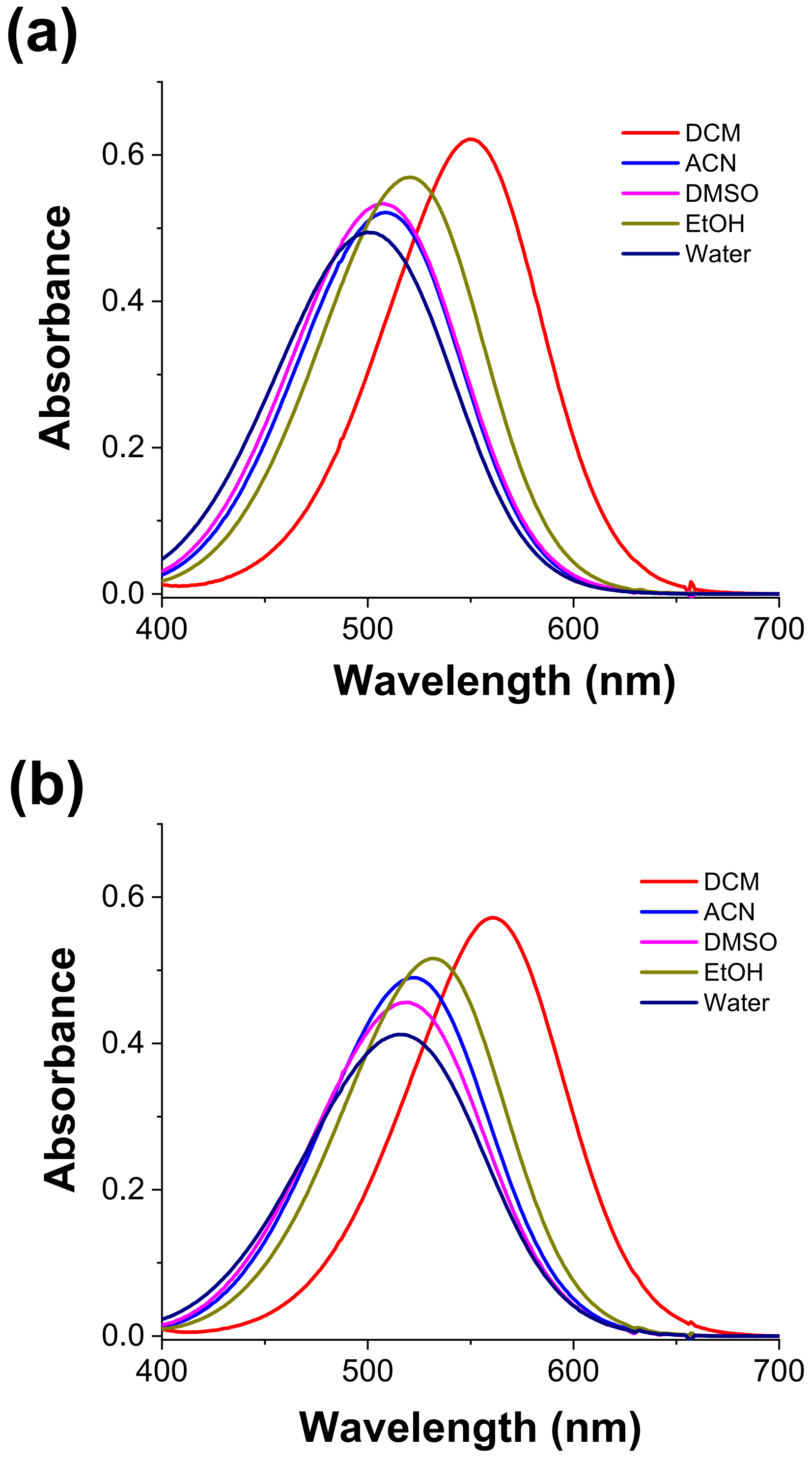

Figure S6 Absorbance of **3**a (a) and **3b** (b) (1 X 10^{-5} M) in different solvents at room temperature.

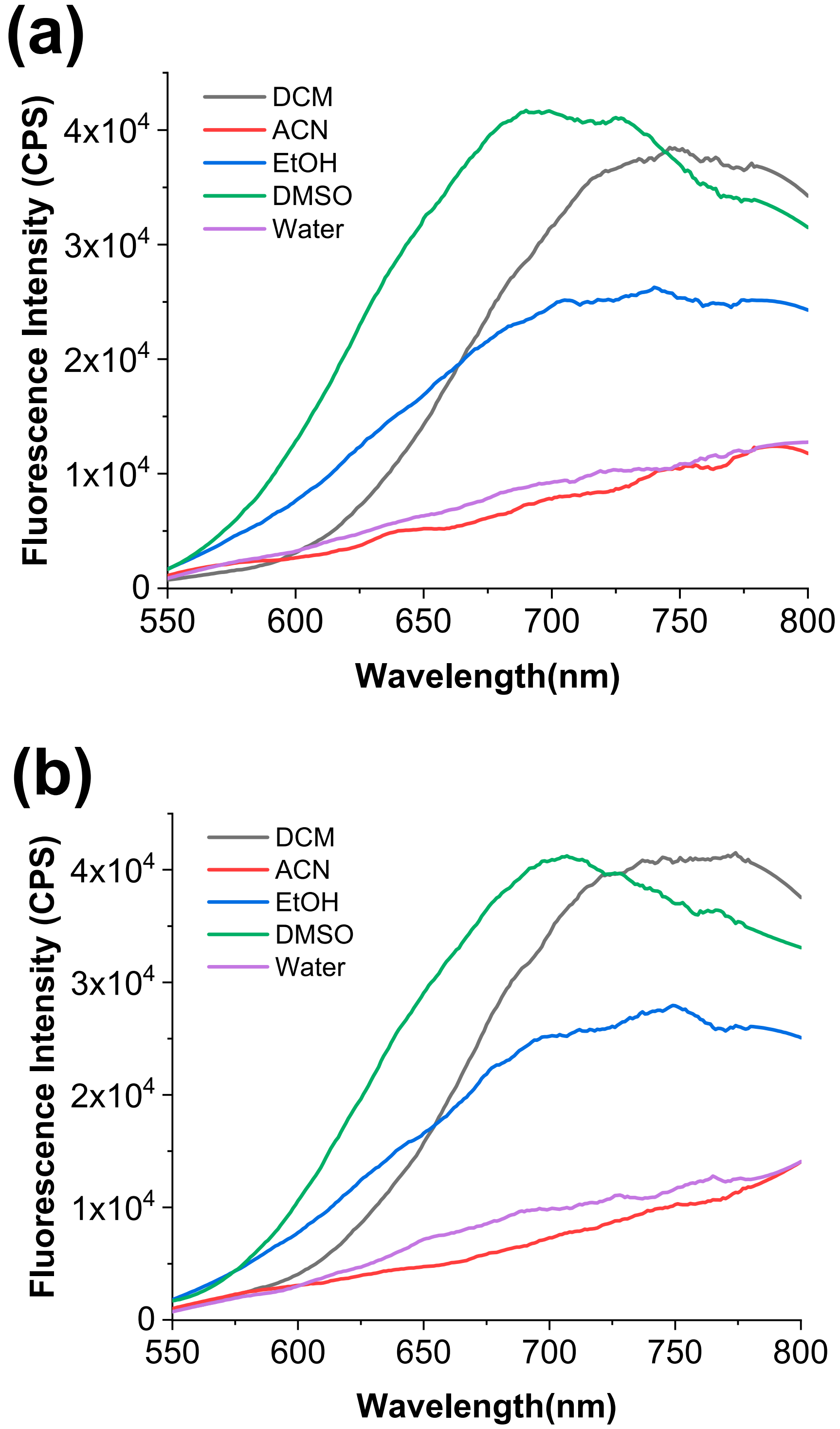

Figure S7 Fluorescence emission of **3a** (a) and **3b** (b) (1 X 10^{-5} M) in different solvents at room temperature. Probes were excited at 520 nm and the emissions were collected form 550 nm to 800 nm.

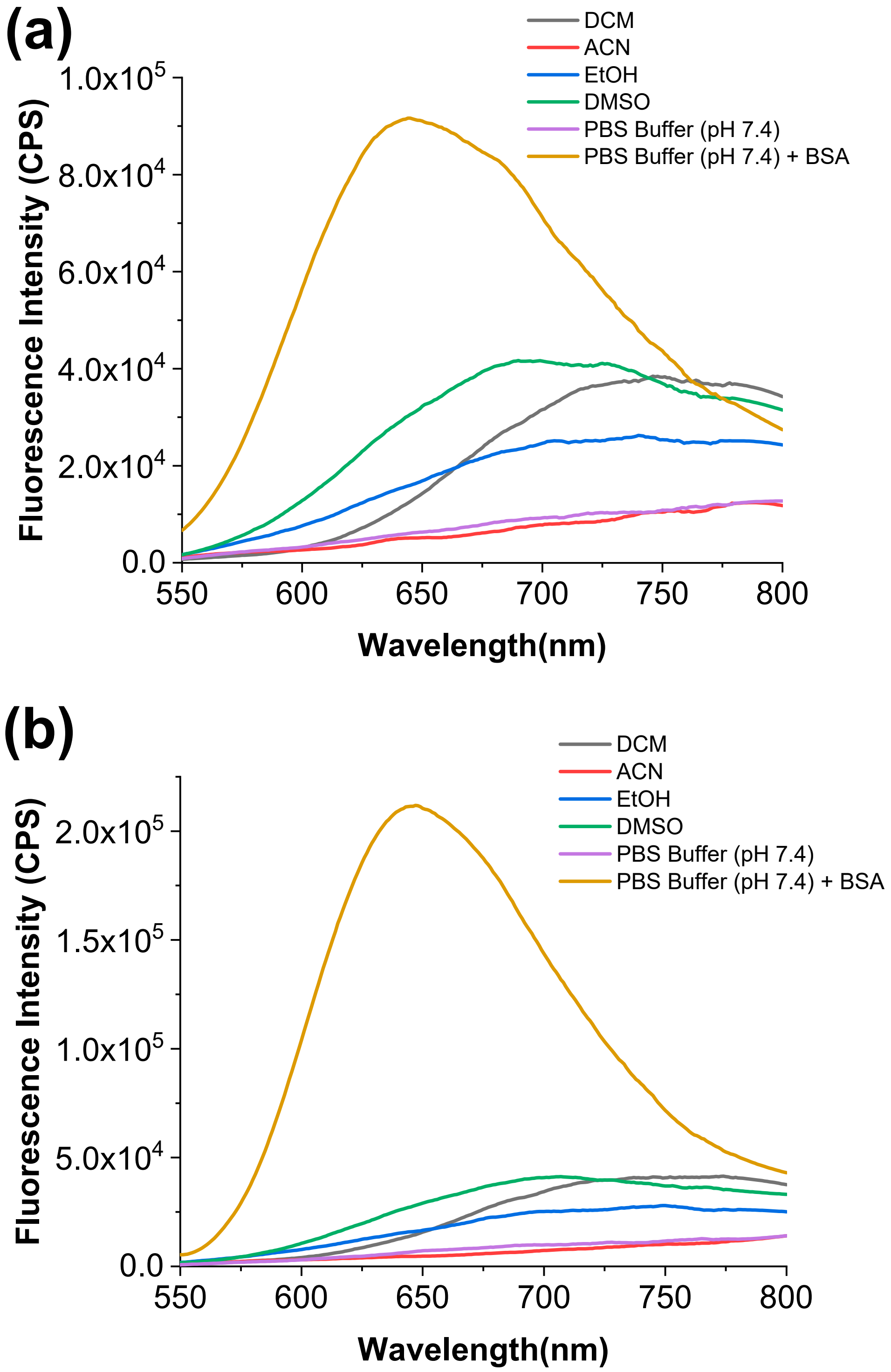

Figure S8 Comparison of the fluorescence emission of **3a** (a) and **3b** (b) (1 X 10^{-5} M) in different solvents with the addition of a 0.50 µL of 10 % BSA into an aqueous solution of the probes (1 X 10^{-5} M in PBS buffer at pH 7.4) at room temperature. Probes were excited at 520 nm and the emissions were collected form 550 nm to 800 nm.

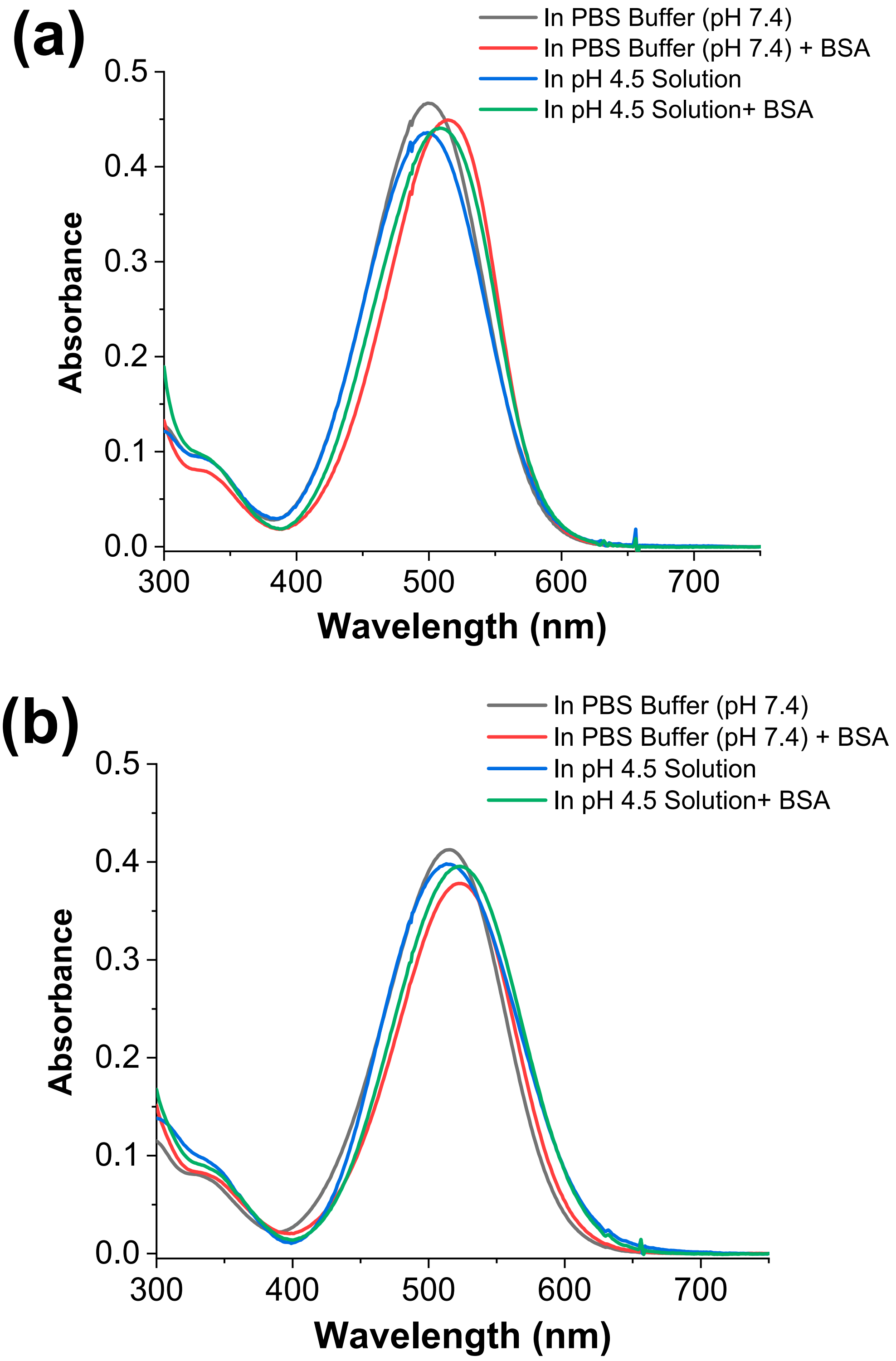

Figure S9 Comparison of absorbance of **3a** (a) and **3b** (b) (1 X 10^{-5} M) in different pH conditions with and without the addition of a 0.50 µL of 10 % BSA at room temperature. Probes were excited at 520 nm and the emissions were collected form 550 nm to 800 nm.

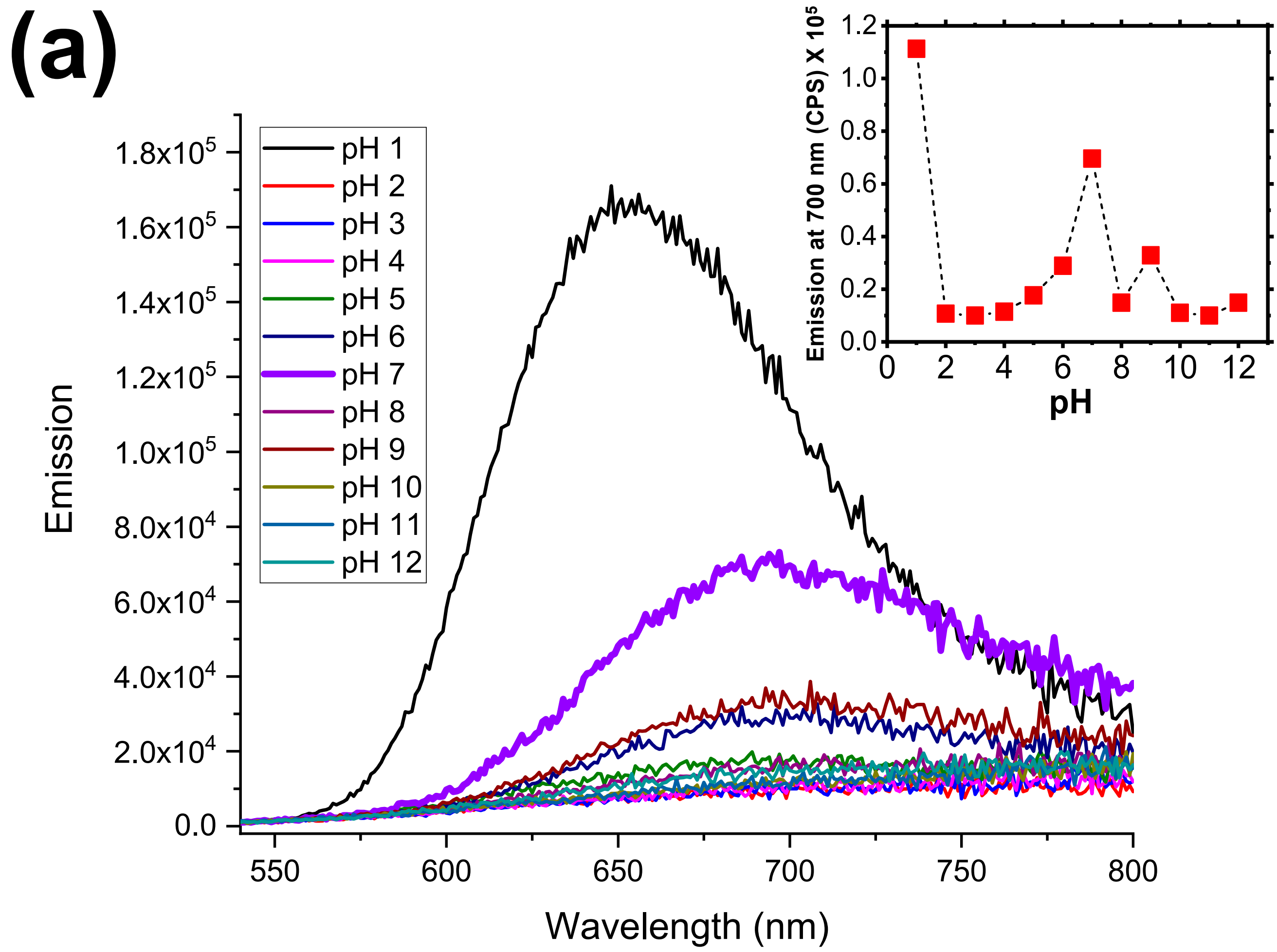

Figure S10.1 The emission spectra recorded for **3a** (1 X 10^{-5} M) in different pH conditions at room temperature. Probes were excited at 510 nm and the emissions were collected form 550 nm to 800 nm.

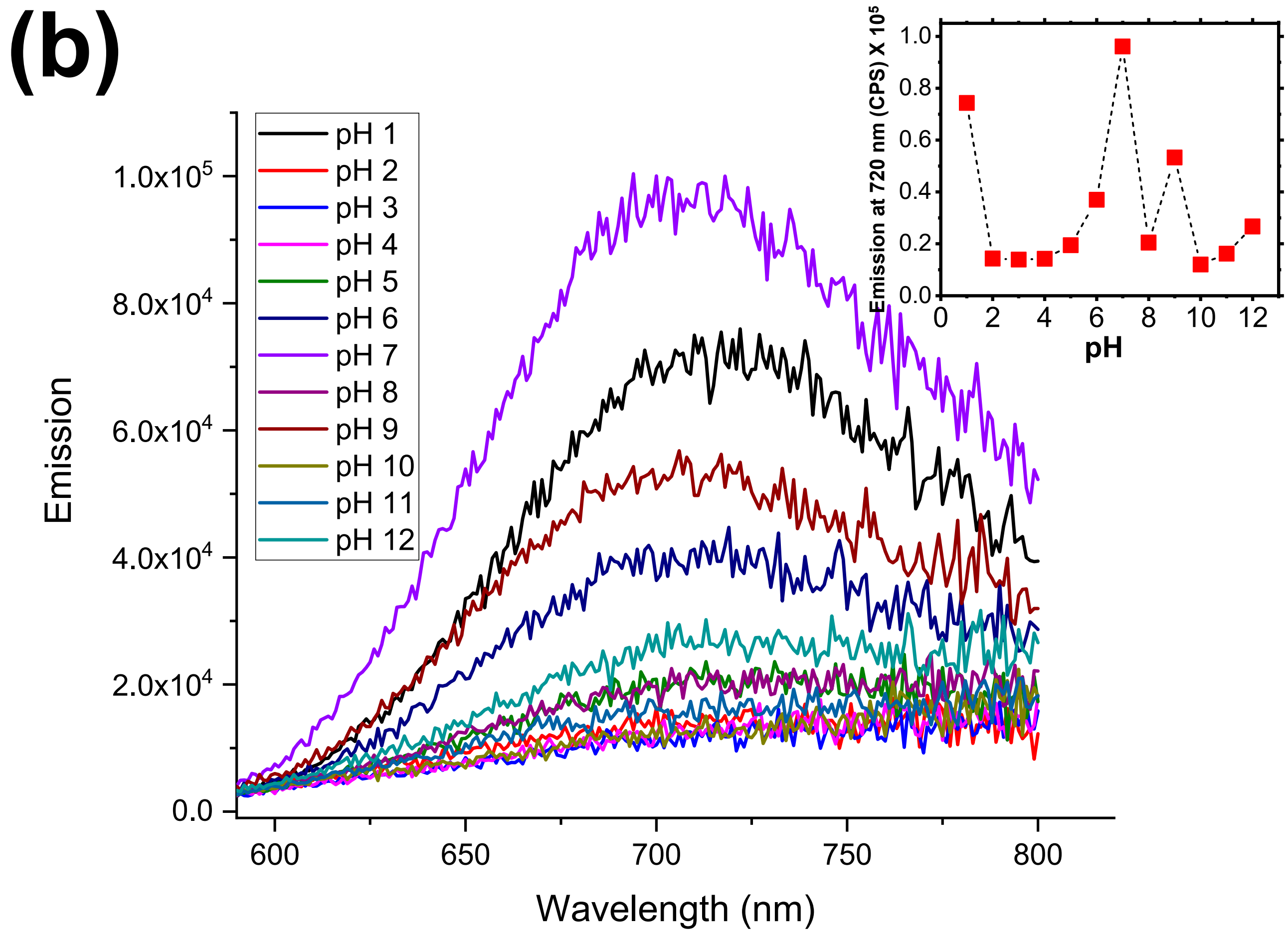

Figure S10.2 The emission spectra recorded for **3b** (1×10^{-5} M) in different pH conditions at room temperature. Probes were excited at 510 nm and the emissions were collected form 550 nm to 800 nm.

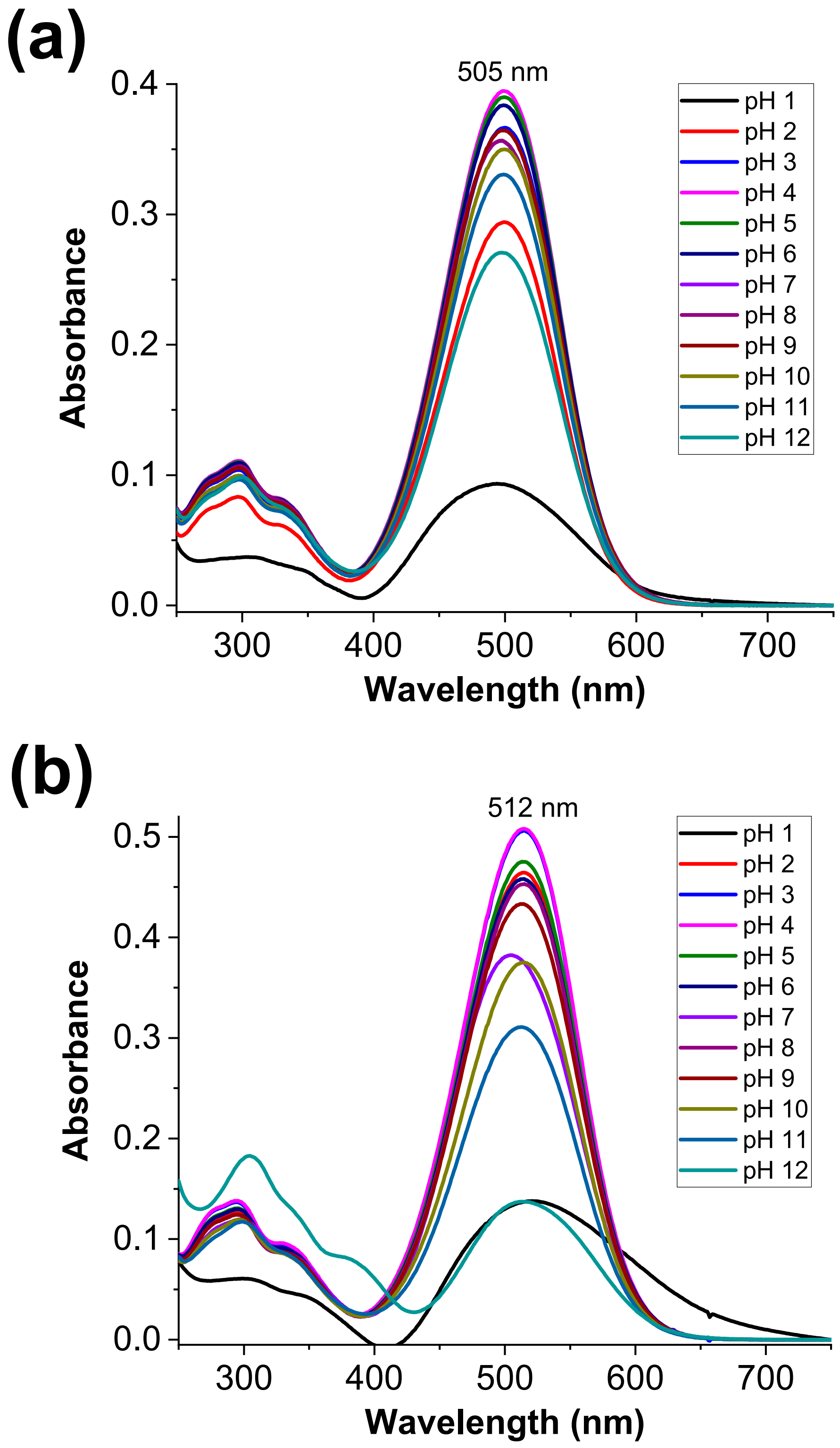

Figure S11 The absorbance spectra recorded for **3a** (a) and **3b** (b) (1×10^{-5} M) in different pH conditions at room temperature.

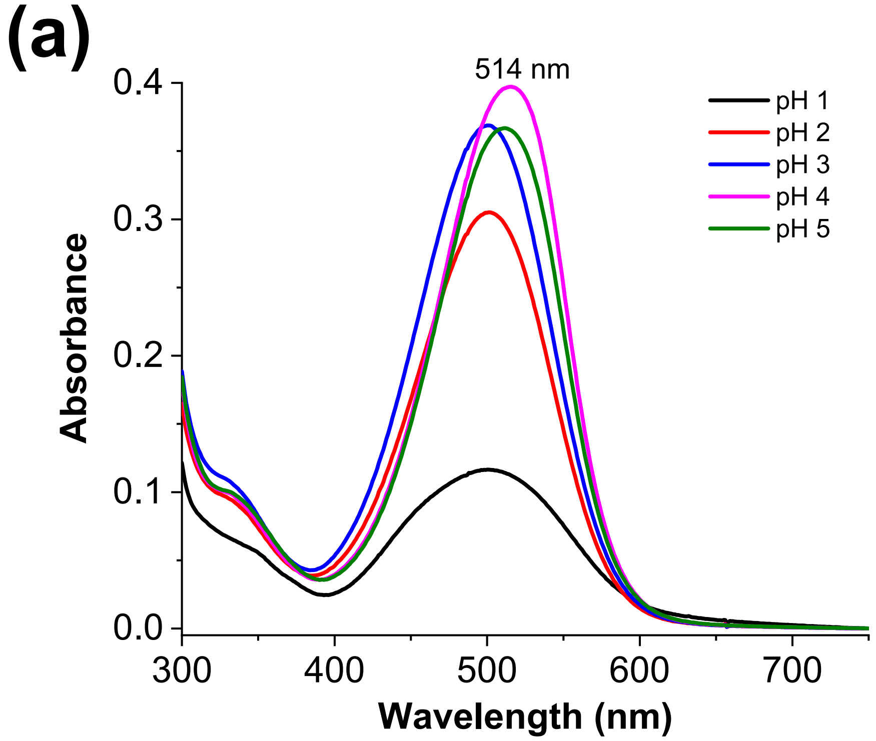

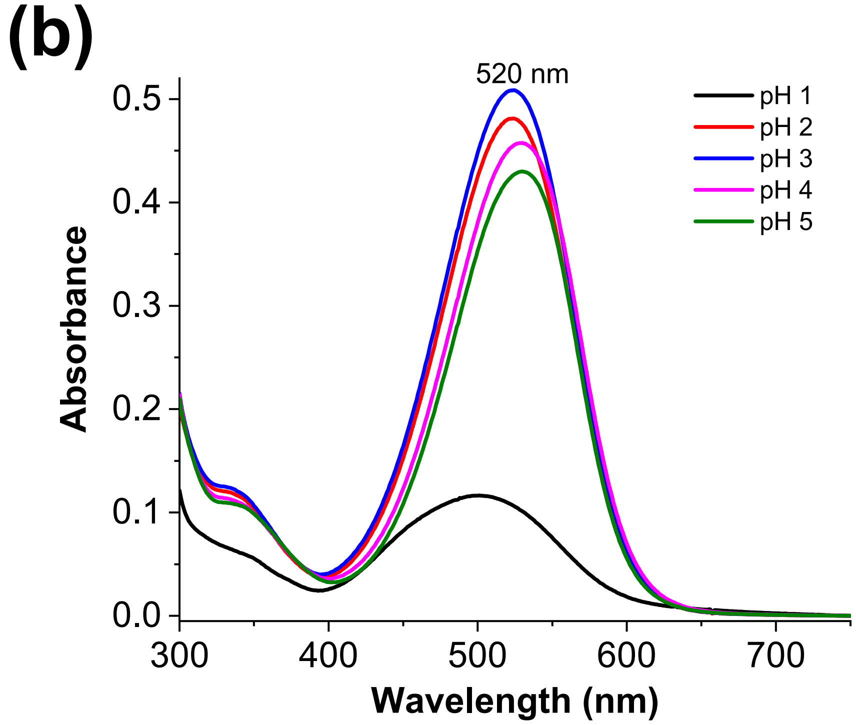

Figure S12 The absorbance spectra recorded for **3a** (a) and **3b** (b) (1 X 10^{-5} M) in different pH conditions at room temperature upon addition of 10 µL of 10% HSA.

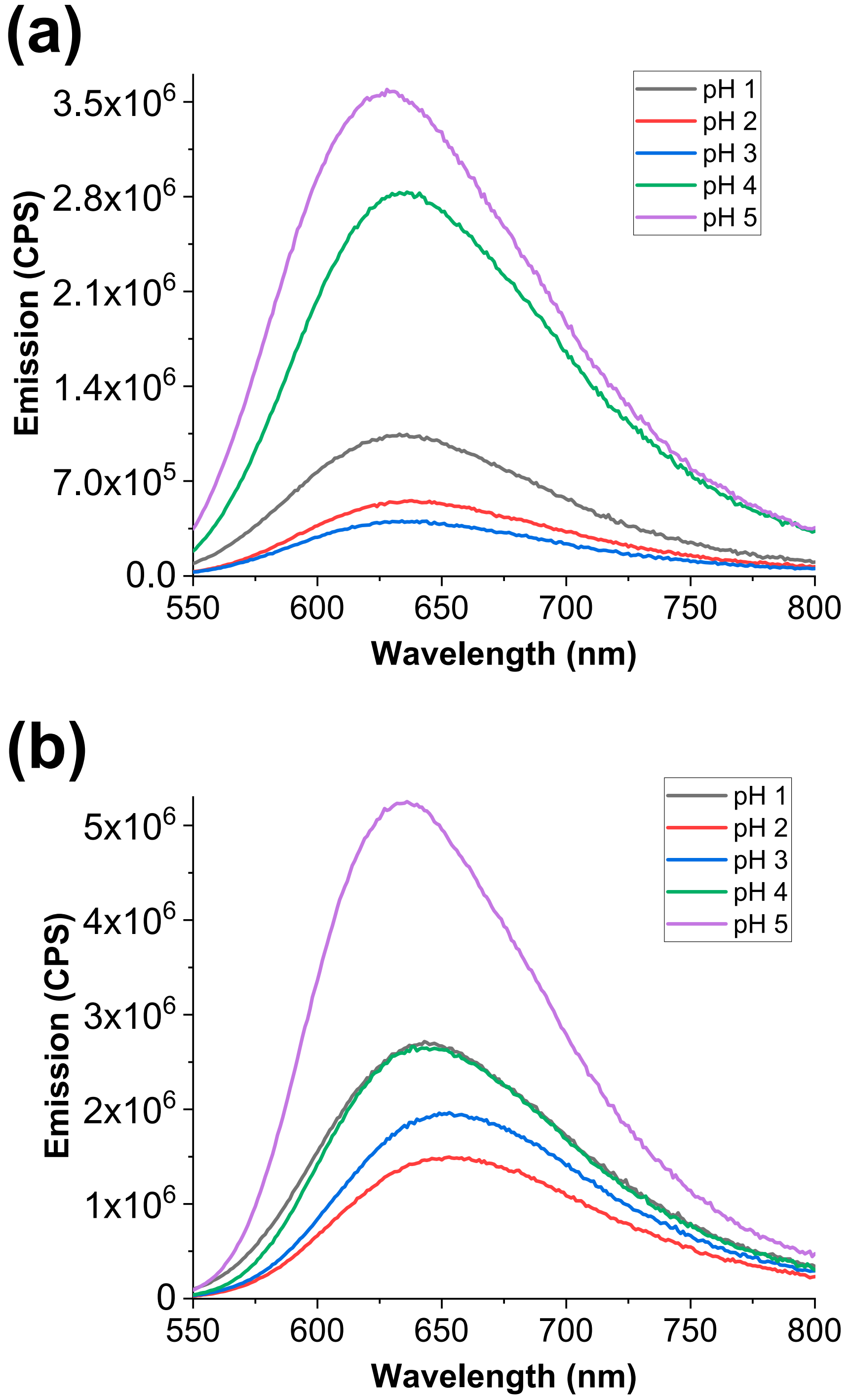

Figure S13 The emission spectra recorded for **3a** (a) and **3b** (b) (1 X 10^{-5} M) in different pH conditions at room temperature upon addition of 10 μL of 10% HSA. Probes were excited at 510 nm and the emissions were collected form 550 nm to 800 nm.

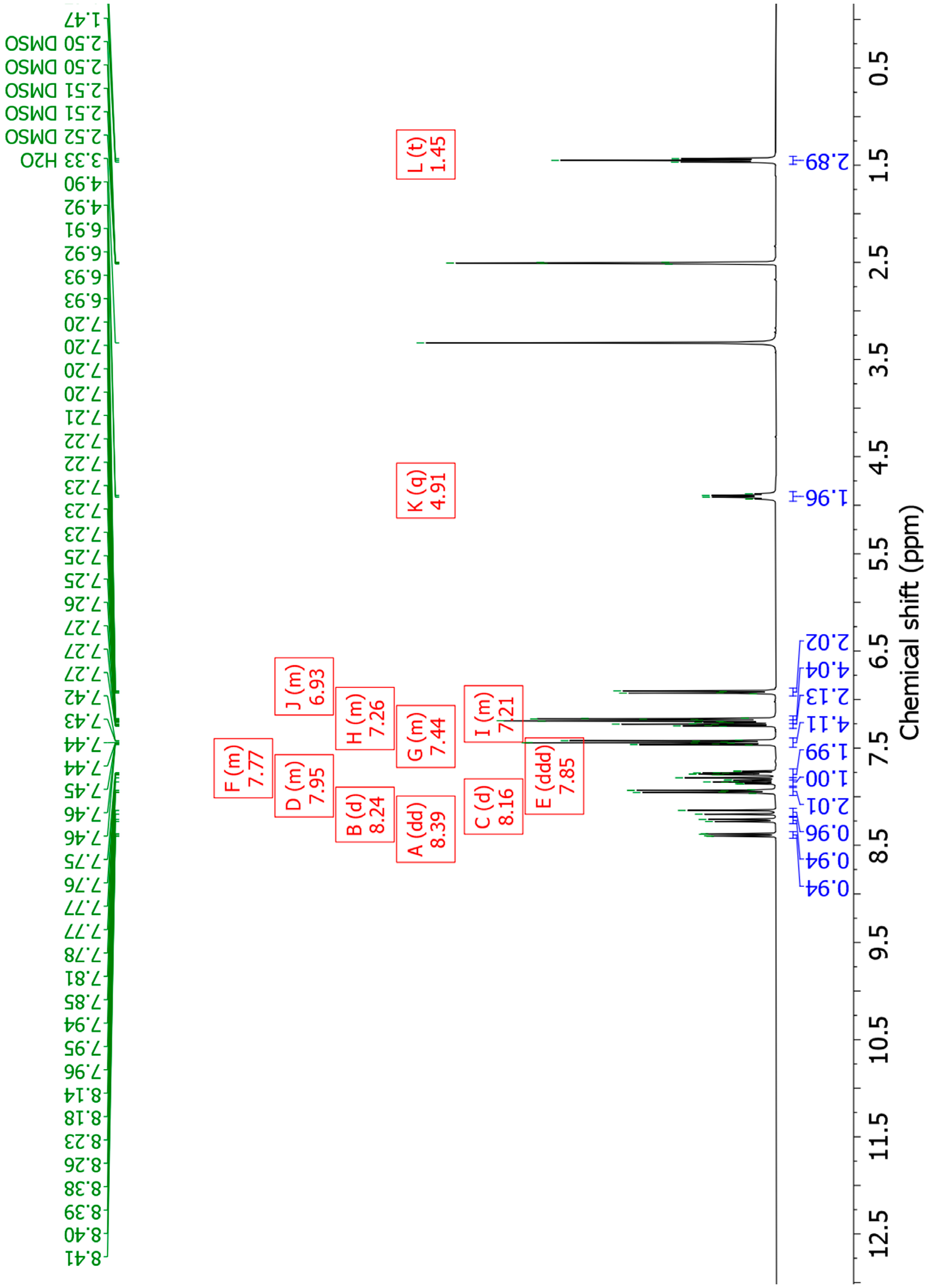

Figure S14.1 ^{1}H NMR spectrum of probe **3a** in DMSO-*d6* (400 MHz).

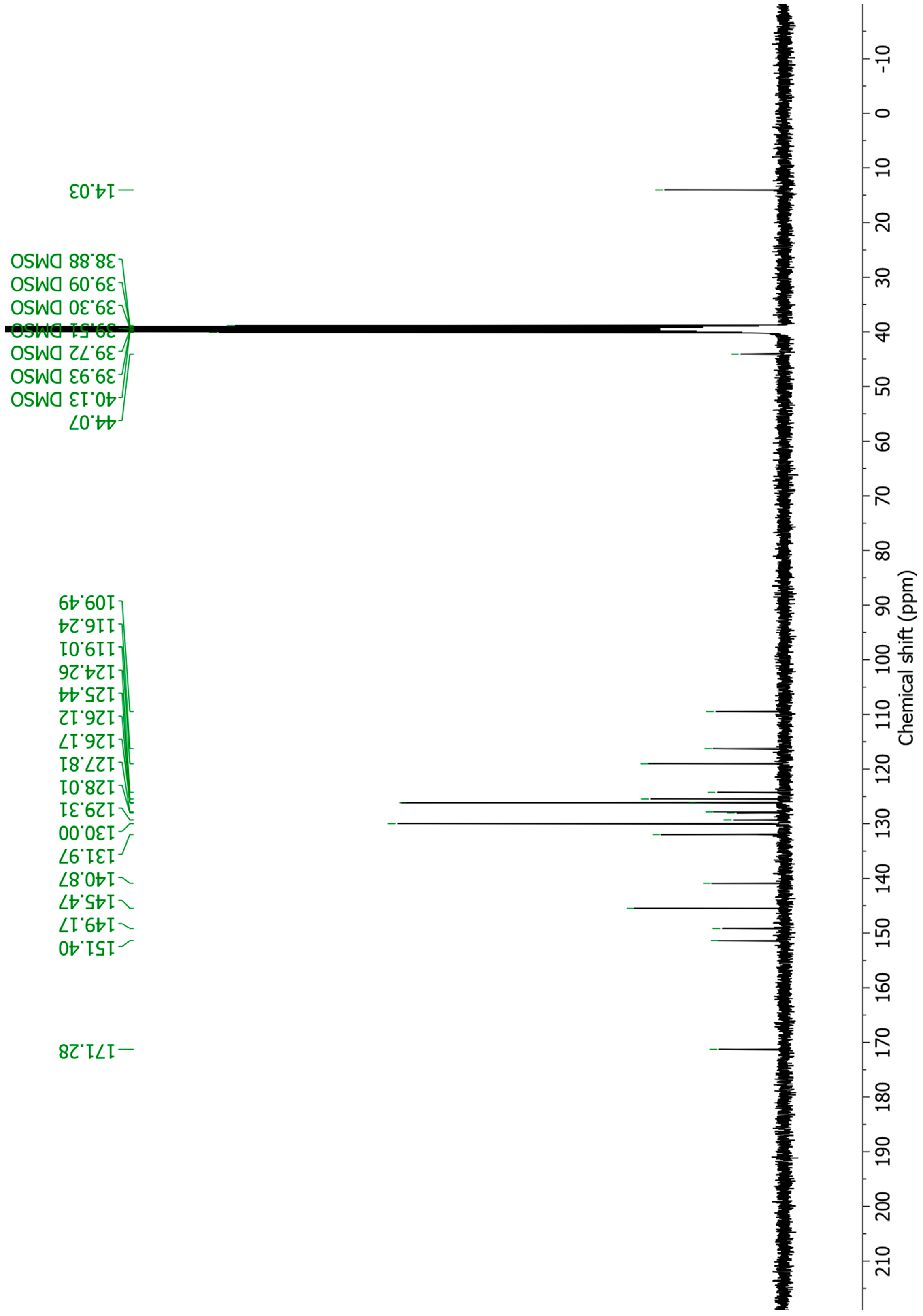

Figure S14.2 ^{13}C NMR spectrum of probe **3a** in DMSO-*d6* (400 MHz).

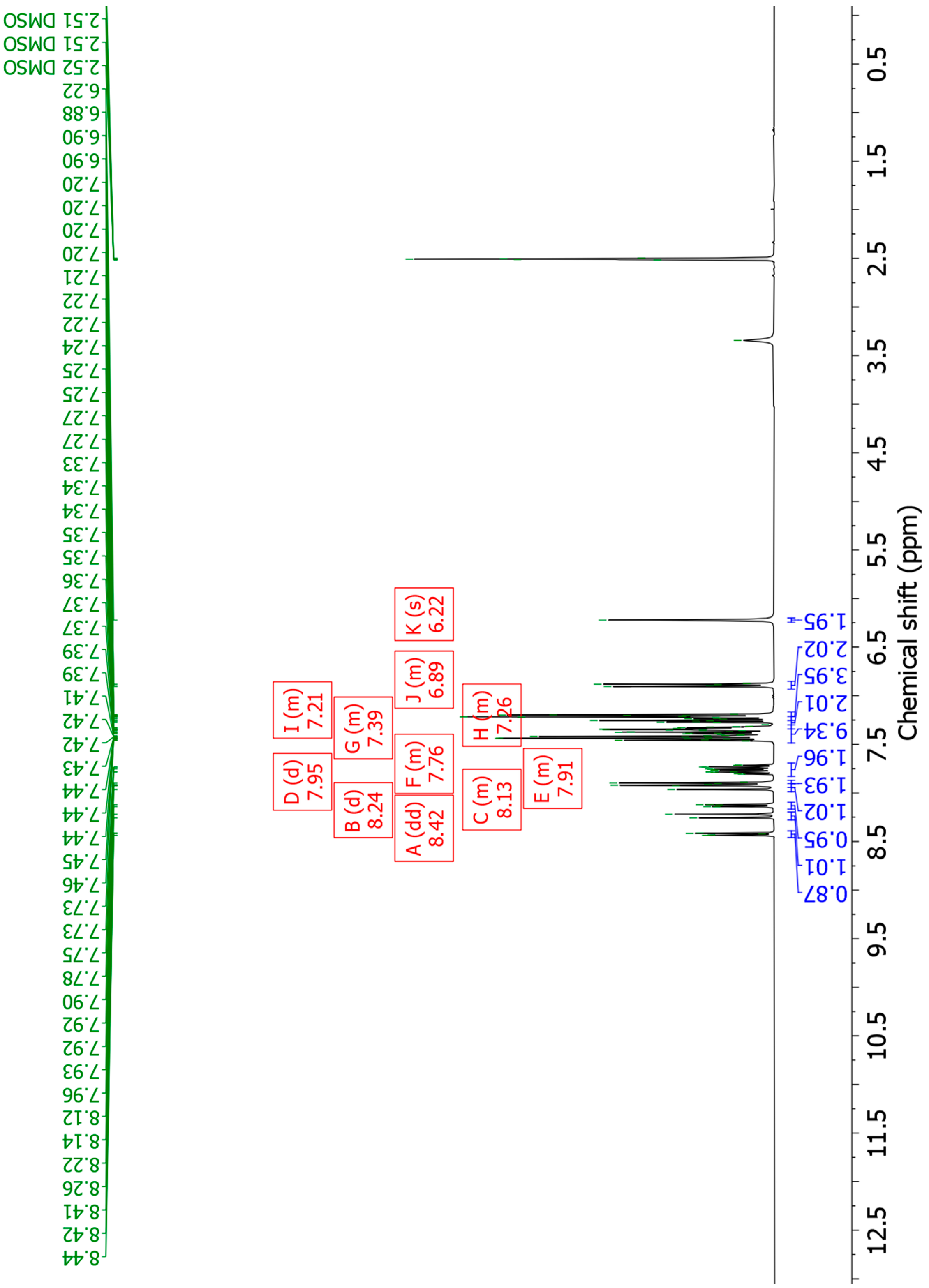

Figure S14.3 ^{1}H NMR spectrum of probe **3b** in DMSO-*d6* (400 MHz).

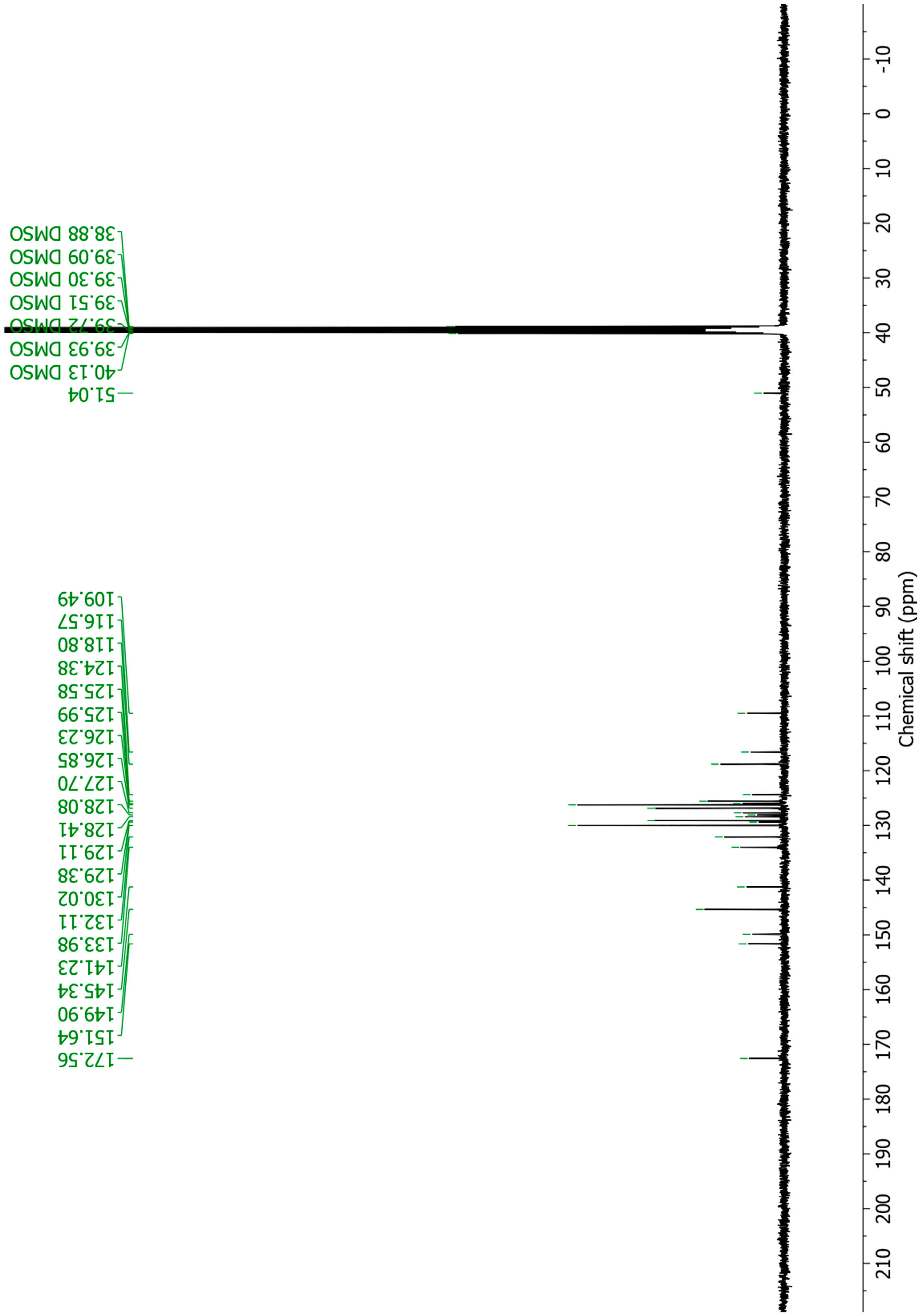

Figure S14.4 ^{13}C NMR spectrum of probe **3b** in DMSO-*d6* (400 MHz).

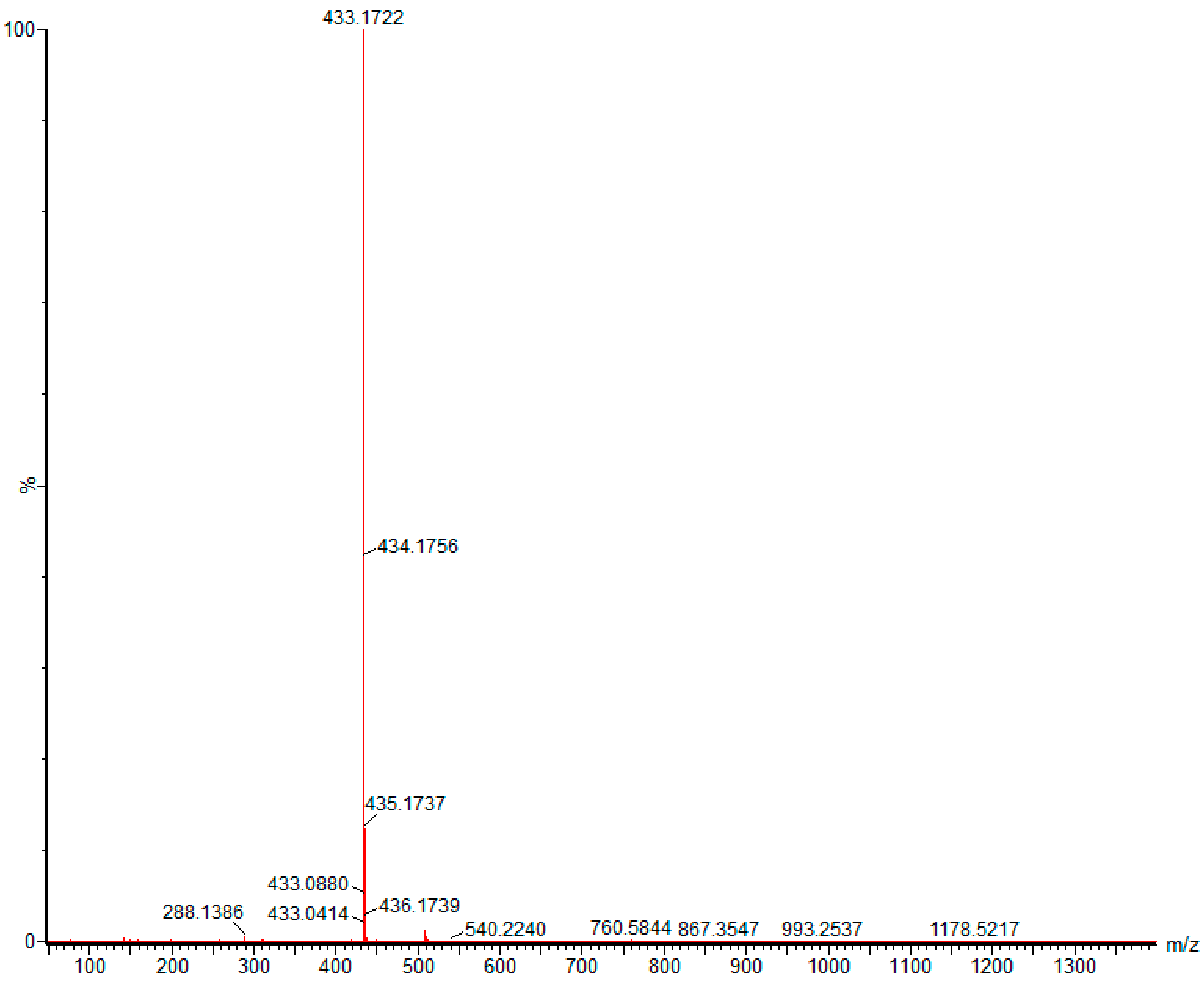

Figure S14.5 High-resolution mass spectra (TOF MS ES+) of **3a** (Molecular Formula: $[C_{29}H_{25}N_2S]^+$). The calculated error is -2.54 ppm

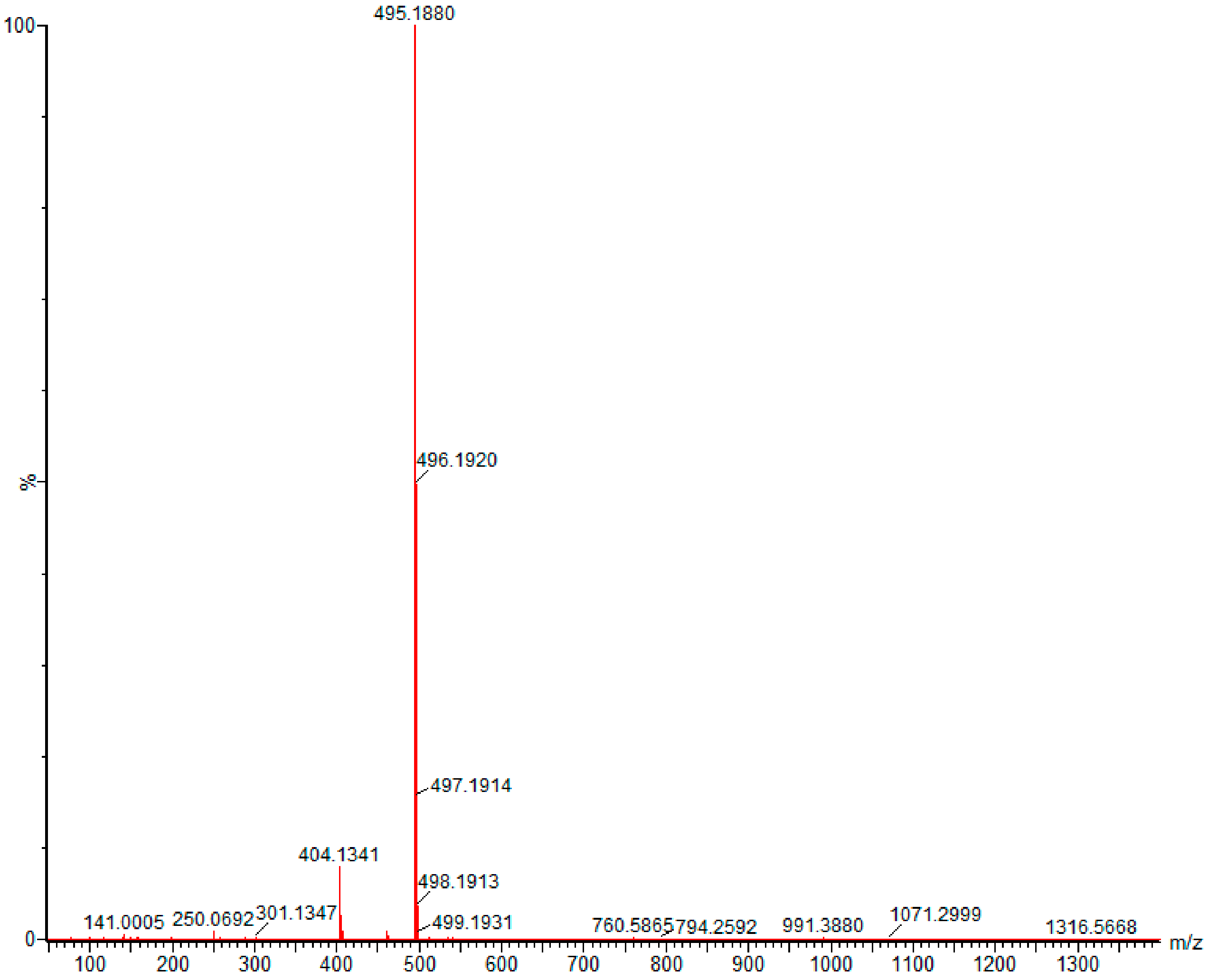

Figure S14.6 High-resolution mass spectra (TOF MS ES+) of **3b** (Molecular Formula: $[C_{34}H_{27}N_2S]^+$). The calculated error is -1.82 ppm.
